# Molecular Traits and Functional Analysis of the CLAVATA3/Endosperm Surrounding Region-Related Small Signaling Peptides in Three Species of *Gossypium* Genus

**DOI:** 10.3389/fpls.2021.671626

**Published:** 2021-06-04

**Authors:** Huan Lin, Wei Wang, Xiugui Chen, Zhenting Sun, Xiulan Han, Shuai Wang, Yan Li, Wuwei Ye, Zujun Yin

**Affiliations:** ^1^Research Base, Zhengzhou University, State Key Laboratory of Cotton Biology, Institute of Cotton Research of Chinese Academy of Agricultural Sciences, Anyang, China; ^2^State Key Laboratory of Crop Biology, College of Agronomy, Shandong Agricultural University, Tai'an, China

**Keywords:** CLE peptide, molecular traits, functional analysis, *Gossypium hirsutum*, *Gossypium raimondii*, *Gossypium arboreum*

## Abstract

The CLAVATA3/endosperm surrounding region-related (CLE) small peptides are a group of C-terminally encoded and post-translationally modified signal molecules involved in regulating the growth and development of various plants. However, the function and evolution of these peptides have so far remained elusive in cotton. In this study, 55, 56, and 86 *CLE* genes were identified in the *Gossypium raimondii, Gossypium arboreum*, and *Gossypium hirsutum* genomes, respectively, and all members were divided into seven groups. These groups were distinctly different in their protein characteristics, gene structures, conserved motifs, and multiple sequence alignment. Whole genome or segmental duplications played a significant role in the expansion of the *CLE* family in cotton, and experienced purifying selection during the long evolutionary process in cotton. *C*is-acting regulatory elements and transcript profiling revealed that the *CLE* genes of cotton exist in different tissues, developmental stages, and respond to abiotic stresses. Protein properties, structure prediction, protein interaction network prediction of GhCLE2, GhCLE33.2, and GhCLE28.1 peptides were, respectively, analyzed. In addition, the overexpression of *GhCLE2, GhCLE33.2*, or *GhCLE28.1* in *Arabidopsis*, respectively, resulted in a distinctive shrub-like dwarf plant, slightly purple leaves, large rosettes with large malformed leaves, and lack of reproductive growth. This study provides important insights into the evolution of cotton *CLEs* and delineates the functional conservatism and divergence of *CLE* genes in the growth and development of cotton.

## Introduction

CLAVATA3/embryo surrounding region (CLE) peptides are one of the largest groups of post-translationally modified and short-secreted signaling peptides. They are post-translationally cleaved and then modified from their corresponding pre-propeptides to produce a ligand containing 12–13 amino acids. These peptides function by binding to a corresponding taxon of receptors and are primarily involved in the growth and development of plant meristematic tissues, including shoot apical meristem (SAM) (Fletcher et al., 1999), root apical meristem (RAM) (Stahl et al., 2009), vasculature (Etchells et al., 2015), and legume nodule meristem (Okamoto et al., 2009; Mortier et al., 2010; Lim et al., 2011), as well as respond to environmental stimuli (Wang et al., 2015). The *CLE* gene family was named following the first discovery of *AtCLAVATA3 (CLV3)* in *Arabidopsis thaliana* and *ESR* in *Zea mays* and it is considered to be structurally conserved group, *CLV3* and *ESR* are similar in structure and function but unrelated. The protein sequences possess a small, conserved region of 14 amino acids (Opsahl-Ferstad et al., 1997; Cock and McCormick, 2001) and are expressed specifically in the embryo surrounding region (*ESR*) of the endosperm in *Zea* (Opsahl-Ferstad et al., 1997). Different *ESR* members exhibit varying levels of expression in the same region of the maize endosperm (Bonello et al., 2000).

Most plant CLE protein sequences are characterized by several common structural motifs, consisting of a signal motif of 45–90 nucleotides in length located at the N-terminus, a central changeable region ranging from 120 to 240 nucleotides (40–90 amino acids), and a highly conserved functional domain near the C-terminus (a conserved sequence of 14 amino acids: KRXVPXGPNPLHNR) (Cock and McCormick, 2001; Hastwell et al., 2015b, 2017a). Variable regions in CLE protein are essential to their function, and can be used to further clarify the differences in the family members of various species and subfamilies (Ni and Clark, 2006). In addition, some *CLE* genes encode an extra C-terminal domain (1–150 amino acids), not conserved among non-orthologous proteins, called a C-terminal extension. Members containing this type of domain have been found in some plant species, but little research has been done on their processing (Kinoshita et al., 2007; Oelkers et al., 2008; Strabala et al., 2014). In recent years, CLE-like peptides have gradually been found to share a similar structure but display unrelated activity in the functional domain (Meng et al., 2012). The tracheary element differentiation inhibitory factor (TDIF) is a *CLE*-like peptide, in which the functional domain consists of 12 amino acids containing two hydroxyproline residues (Hirakawa et al., 2010). The conserved functional domains of AtCLE41 and AtCLE44 are the same as that of TDIF. They interact with the TDIF receptor/phloem intercalated with xylem (LRR-RLK TDR/PXY) membrane protein kinase and are involved in promoting the proliferation of procambial cells and suppression of xylem differentiation (Hirakawa et al., 2008, 2010).

The biological functions of some *CLE* genes have been well-studied and characterized in model plants, such as *Arabidopsis* and rice. For example, *CLV3* plays a critical role in SAM to regulate stem cell homeostasis (Fletcher et al., 1999). Overexpression of *CLV3* inhibits organ initiation after the appearance of the first leaves, while *CLV3* mutants display the opposite phenotype, including the build-up of stem cells in the center of shoot and floral meristems (Brand et al., 2000). In rice, *floral organ number 4* (*FON4*), encodes a putative ortholog of *AtCLV3*, which is mainly expressed in a small group of cells at the apex of the SAMs. The *FON4* mutants display abnormal enlargement of the embryonic and vegetative SAMs as well they increase in inflorescences and floral meristems (Chu et al., 2006). The ectopic expression of 18 different *CLE* genes (*CLV3, CLE2, CLE3, CLE4, CLE5, CLE6, CLE7, CLE9, CLE10, CLE11, CLE13, CLE18, CLE19, CLE21, CLE25, CLE26, CLE42*, and *CLE44*) in *Arabidopsis* resulted in similar levels of premature mortality or phenotypes with developmental timing delays (Strabala et al., 2006). *Arabidopsis CLE* genes that are involved in root development have also been fully described. For example, *AtCLE40*, encoding a peptide with close structural similarity to *AtCLV3*, plays an important role in the control of root tip movement and root length (Hobe et al., 2003). Furthermore, *AtCLE1, 3, 4*, and *7* are induced mainly in root pericycle cells under nitrogen deficiency, and overexpression of these genes suppresses the growth of lateral root primordia (Araya et al., 2014). *AtCLE8* is expressed in seed embryos and endosperm and controls the expression of *Wuschel-like homeobox 8* (*WOX8*), and together *AtCLE8* and *AtWOX8* form a signal transduction pathway involved in seed morphogenesis (Fiume and Fletcher, 2012). *AtCLE41, AtCLE42*, and *AtCLE44* are known as tracheary element differentiation factors, and they control vascular meristematic tissue cell proliferation and differentiation by interacting with their receptor PXY/TDR (Hirakawa et al., 2008). However, many *CLE* gene family members have not yet been fully identified and their roles remained unclarified.

As a vital economic crop, cotton provides natural fibers to the world textile industry. *Gossypium*, the heterotetraploid, is the most widely planted cultivar that originated from a natural hybridization between the primitive *G. arboreum* (A-genome species) and native *G. raimondii* (D-genome species) (Hu et al., 2019). The biological mechanisms of CLE signal peptides in some simple plants have been well-studied. However, the evolution and functional differentiation of cotton *CLE* remain unknown. In this study, the members of the *CLE* family were identified genome-wide from three cotton genomes, and characterized for their sequence features, phylogenetic relationships, *cis*-elements, protein interaction, expression levels in tissue-specific, and in response to different abiotic stresses and functional verification. These results provide a treasurable resource to further dig out other functions and molecular mechanisms of the *CLEs* in cotton.

## Materials and Methods

### Sequence Search and Identification of Cotton *CLE* Genes

To identify the members of the *CLE* genes family in *G. raimondii, G. arboreum*, and *G. hirsutum*, protein sequences from *A. rabidopsis* and *Populus trichocarpa* in the published literature were used as query sequences (Han et al., 2016). They were downloaded from the *Arabidopsis* Information Resource (http://www.Arabidopsis.org) and *Populus* genome (http://www.phytozome.net/), respectively (Goodstein et al., 2012; Reiser et al., 2017). A BLAST (SequenceServer 2.0.) search was performed in the Cotton Gene database (http://www.cottongen.org) using BLASTP (Version 2.2) with e-value cutoff of 1e^−10^ (Yu et al., 2014; Li and Lu, 2019). Moreover, HMMER (version 2.41.1) model construction was also adopted for further retrieval (Potter et al., 2018). Candidate proteins without a conserved CLE motif (KRXVPXGPNPLHNR) were removed.

### Analysis of Protein Features and Subcellular Localization Prediction

Using the website of CELLO v2.5 (http://cello.life.nctu.edu.tw/), the subcellular localization of the CLEs was predicted (Yu et al., 2004), and the molecular weight (Mw) and isoelectric point (pI) of the candidate members were calculated according to the ExPASy server (http://web.expasy.org/compute_pi/) (Bjellqvist et al., 1994; Wilkins et al., 1999). The CLE signal peptide cleavage sites were predicted using SignalP-5.0 (http://www.cbs.dtu.dk/services/SignalP/) (Almagro Armenteros et al., 2019).

### Multiple Sequence Alignment Analysis and Construction of Phylogenetic Trees

ClustalX 2.0 was used to perform multi-sequence alignment (Thompson et al., 1997). The full-length *CLE* sequences were aligned using ClustalW and then an unrooted phylogenetic tree was constructed using MEGA 7.0 with the neighbor-joining (NJ) method, which was further verified using the maximum likelihood (ML) method (Kumar et al., 2016). To support the presumed evolutionary relationships, the bootstrap method was used with 1,000 replicates. Weblogo3 (http://weblogo.threeplusone.com/) was used to predict the CLE functional domains and to characterize the protein features (Crooks et al., 2004).

### Analysis of Chromosomal Locations, Gene Structures, and the Conservative Domain

The CDS and genomic sequences of the *CLE* genes in *G. raimondii, G. arboreum*, and *G. hirsutum* were searched in the Cotton Functional Genomics Database, using the known gene ID (https://cottonfgd.org/about/download.html) (Zhu et al., 2017). TBtools (https://github.com/CJ-Chen/TBtools) was employed locally to map the *CLE* genes on the chromosomes (Chen et al., 2020). The structure of the *CLE* genes was displayed using Gene Structure Display Server 2.0 (http://gsds.cbi.pku.edu.cn), (Hu et al., 2015). The conserved sequence and number of conserved motifs were analyzed using the MEME Suite 5.3.3 (http://meme-suite.org/) (Bailey et al., 2009). The maximum number of conservative motifs was set to five, and then each subfamily was predicted one by one.

### Analysis of Collinearity, Duplication, and Ka/Ks Values

Chromosomal location and gene duplication events were analyzed using the Advanced Circos of TBtools (https://github.com/CJ-Chen/TBtools). To exhibit segmentally duplicated pairs and orthologous pairs of *CLE* genes, the Multiple Synteny Plotter TBtools was used to draw collinearity maps (Chen et al., 2020). The Ka (non-synonymous substitution rate)/Ks (synonymous substitution rate) ratios of the *CLE* genes were calculated using KaKs_calculator 2.0 (Wang et al., 2010).

### Analysis of *cis*-Acting Elements of Promoter Regions and Protein Interaction Analysis

The promoter sequences (2,000 bp upstream of the initiation codon “ATG”) of 86 gene members were filtered from the *G. hirsutum* genome (including GFF3 and FASTA sequence files) by TBtools. Putative *cis*-acting elements were identified using the online Plant CARE server (http://bioinformatics.psb.ugent.be/webtools/plantcare/html/search_CARE.html) and then visualized with TBtools (Lescot et al., 2002).

**Figure 1:**
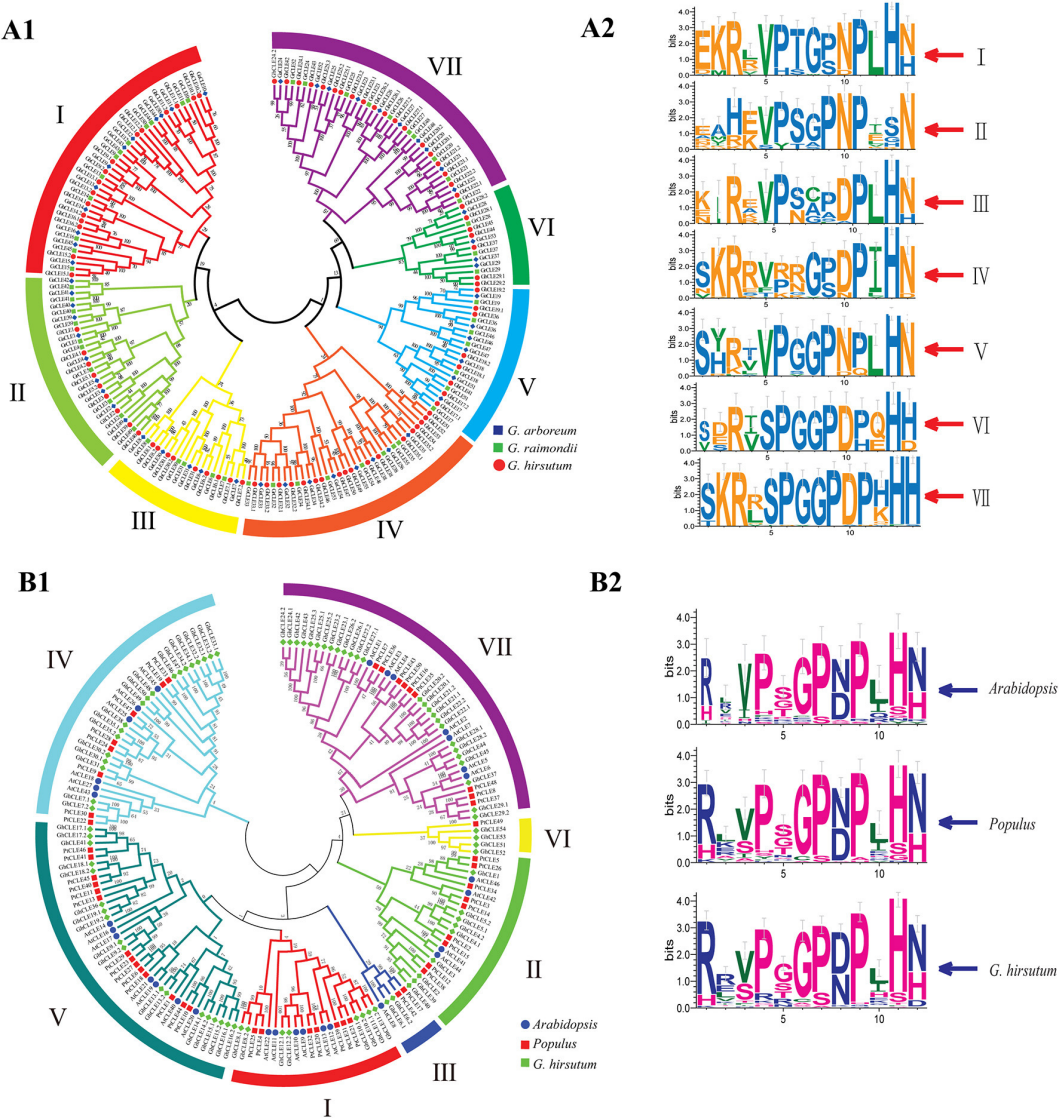
Phylogenetic trees were established using MEGA7 with the neighbor-joining (NJ) method. **(A1)** Phylogenetic tree of CLEs among *G. raimondii, G. arboreum*, and *G. hirsutum*. **(A2)** The weblogo represents CLE motifs (14 amino acids) of seven groups. **(B1)** Phylogenetic tree of CLEs among *G. hirsutum, Arabidopsis*, and *Populus*. **(B2)** The weblogo represents CLE motifs of *Arabidopsis, poplar*, and *G.hirsutum* (12 conserved amino acids).

The secondary structure using PHYRE2 Protein Fold Recognition Server (http://www.sbg.bio.ic.ac.uk/phyre2/html/) (Kelley et al., 2015), hydrophobicity/hydrophilicity using ProtScale (https://web.expasy.org/protscale/) (Wilkins et al., 1999), signal peptide using the SignalP-5.0 Server (http://www.cbs.dtu.dk/services/SignalP/) (Almagro Armenteros et al., 2019), and transmembrane domain using the TMHMM Server v. 2.0 (http://www.cbs.dtu.dk/services/TMHMM/) (Chen et al., 2003) of GhCLE2, GhCLE33.2, and GhCLE28.1 peptides were predicted, respectively. Interaction network analysis was performed with STRING (Version 11.0) (https://string-db.org/cgi/input.pl) (Szklarczyk et al., 2019) on the foundation of the homologous proteins in *Arabidopsis* (Figure 1B1).

### Analysis of the Expression Patterns of Cotton *CLE* Genes

The RNA sequencing (RNA-seq) data of the *CLE* genes of *G. hirsutum*, in different tissues and under three abiotic stresses were downloaded from the NCBI Sequence Reading Archive (SRA: PRJNA248163). The expression pattern analyses were performed in roots, stems, leaves, petals, pistils, and stamens from intact mature flowers of *G. hirsutum* and different development stages of ovules. In addition, expression patterns were assessed under stresses including heat, drought, and salt treatments after 0, 1, 3, 6, and 12 h. The expression levels of the *GhCLEs* were calculated from fragments per kilobase million (FPKM) values and the expressed genes were identified using the default empirical abundance threshold of FPKM > 1 (Hart et al., 2013).

### Plant Materials and Treatments

The cotton variety, TM-1 was provided by the Germplasm Bank of Cotton Research Institute, Chinese Academy of Agricultural Sciences. When the cotton seedlings had grown to three euphylla periods in seasonable growth conditions, the roots were irrigated with 20% polyethylene glycol 6000 (PEG6000), 150 mM NaCl solution, and ddH_2_O, respectively (ddH_2_O was used as a mock control). Seeding leaves were harvested after 0, 3, 6, 12, and 24 h under the drought and salt treatment, respectively. The samples were placed in liquid nitrogen immediately and stored at −80°C. Tissue-specific samples were taken from a field in Anyang, China. Roots, stems, leaves, petals, stamens, pistils, and ovules (0, 3, 5, 10, and 15 DPA) were also sampled and stored in the same way for RNA extraction. Three biological replicates of all the samples were used.

#### RNA Extraction and Quantitative Real-Time-PCR Analysis

Total RNA was extracted using an RNA prep Pure Plant Kit (Tiangen, Beijing, China). First-strand complementary DNA (cDNA) was obtained using a PrimeScript RT reagent kit (Perfect Real Time, Takara, Dalian, China). The cDNA samples were mixed with ddH_2_O at a ratio of 1:5 for quantitative real time PCR (qRT-PCR). SYBR Premix ExtaQTM II (TaKaRa, Japan) was used for qRT-PCR analysis on a 7,500 Fast Real-Time PCR system (Applied Biosystems, Inc., California, USA). The *GhCLE* expression levels in specific tissues under normal conditions and leaves under the different stress conditions were calculated using 2^−ΔΔCT^ method. The cotton *GhActin-7-like* gene (Gene Bank ID: AY305733) acted as the endogenous control. All of the qRT-PCR primers were designed using Primer Premier 6.0 software, and are listed in Supplementary Table 9.

#### Vector Construction and Plant Transformation

The ORFs of *GhCLE*2, *GhCLE33.2*, and *GhCLE28.1* were cloned under control of the 35S promoter in the plant expression vector pBI121. The constructed vectors were first transferred into *Agrobacterium tumefaciens* (strain GV3101) and later transferred into wild *Arabidopsis* (Col-0) using the floral-dip method by soaking the inflorescence for 1 min (Clough and Bent, 1998). The T_1_ seeds were screened in 1/2 MS culture medium containing 50 mg/L kanamycin. Homozygous T_2_ lines were obtained by self-pollination and T_2_ transgenic plants were used for phenotype analysis.

## Results

### Identification and Characterization Analysis of CLE Genes in Three Cotton Species

In total 55, 56, and 86 *CLE* genes were identified in *G. ramondii, G. arboreum*, and *G. hirsutum* genomes, respectively. Thirteen genes encoding proteins >15 kDa, and four encoding GhCLEs >20 kDa (*GhCLE51/GhCLE52/GhCLE53/GhCLE54*) were identified. There were 24% CLE proteins that do not have a signal peptide cleavage site, indicating that these may affect the formation of mature peptide. Most of the *CLEs* were localized outside the nucleus of which 65% *CLEs* gene members were located extracellularly (Supplementary Tables 1–3).

The *CLE* genes were unevenly distributed along the chromosomes. In *G. hirsutum*, 86 *GhCLE* members were distributed on all but four of the 26 chromosomes: A02, A13, D03, and D13 (Supplementary Figure 1A). In *G. arboreum*,56 *GaCLE* genes were localized on chromosomes, such as Ga01, Ga03, Ga05, Ga11 (six genes on each), Ga06, Ga10, Ga12 (three genes on each), Ga07 (four genes), Ga08 (nine genes), Ga09 (five genes), and Ga13 (two genes) (Supplementary Figure 1B). In *G. raimondii*, 55 *GrCLE* genes were distributed on all the 13 chromosomes with most residing on chromosome Gr04 (eight *GrCLE* genes). Gr12 contained only one member (Supplementary Figure 1C).

All the *CLE* genes in *G. ramondii, G. arboreum, G. hirsutum*, and *poplar* genomes were divided into seven groups (I–VII), except for *Arabidopsis* (Figure 1 and Supplementary Figure 2). The CLE sequence weblogos were drawn for each group to display the conserved sequences and some differences in the CLE domains among different groups or species were found (Figures 1A2,B2). For example, the last residue of Groups I–V mainly appeared as N (Asn), while the Groups VI and VII were presented as His (H) in cotton. The frequency of the RL (Arg and Leu) motif in *Arabidopsis* and *poplar* was higher than that of the RR motif, while the opposite was found in *G. hirsutum* (Figure 1B2).

All of the *CLE* protein sequences shared three characteristics: an N-terminal signal peptide, a conserved *CLE* motif containing 14 amino acids at or near the C-terminal, and a generally not conserved region between the signal peptide (Supplementary Figure 3). Apart from positions 12 and 13 in Group II and positions eight and nine in Group III, the CLE motifs (at amino acids 5–14) in each group were highly conserved in cotton, with 10 residues remaining almost unvaried. The CLE motifs of Group II lacked conservation of the His residue at position 13, which did not vary in the remaining groups. The Asn residues at position 14 in the Groups I–V were quite conserved, while in the Groups VI–VII, this position was taken by His (Figure 1A1,A2). The cotton CLE domain (12 residues) was highly conserved except 2, 3, 5, and 10 positions, which was similar to that seen in *Arabidopsis* and *poplar* (Figure 1B2).

Most of the *CLE* members did not contain introns, especially Groups V, VI, and VII and the remaining members contained two to three exons, except *GaCLE38*, which consisted of 11 exons and 10 introns. Five conserved domains were predicted in each monophyletic group. The motif map revealed that some genes may have lost or acquired conserved motifs during evolution. For example, *GhCLE52* in Group IV may have lost motif 4 in comparison to its homologous gene, *GhCLE51*, while *GhCLE31* in Group III may have acquired motif three compared to *GrCLE31* and *GaCLE31*. *GhCLE51–GhCLE54*, encode CLE proteins carrying multiple identical motifs, containing 2, 5, 1, and 3 replicates of motifs 1 and 2, 2, 1, and 3 replicates of motif 5, respectively (Supplementary Figure 4).

### Analysis of Collinearity, Duplication, and Ka/Ks Values of the *CLEs* Family

**Figure 2:**
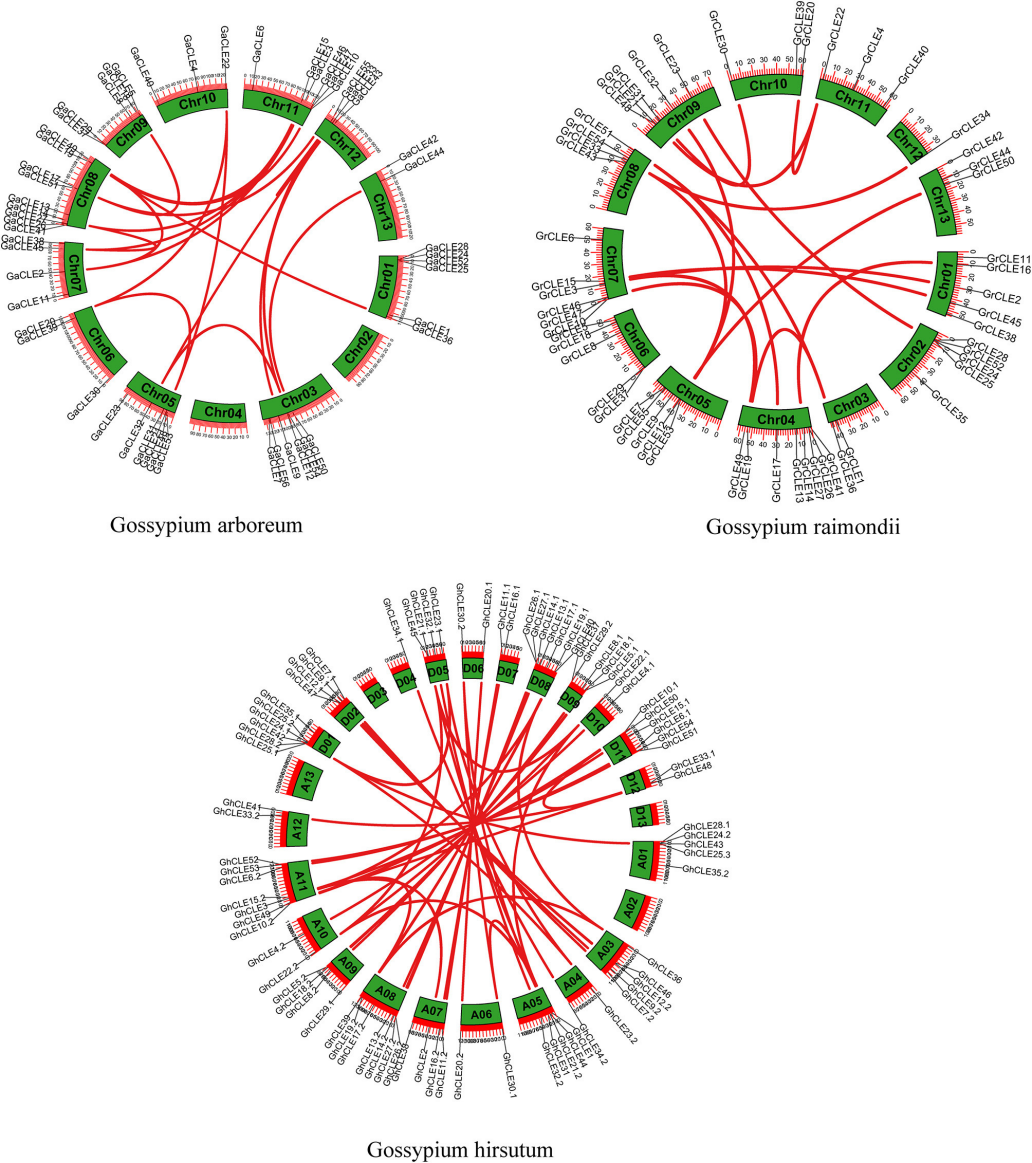
Distribution of *CLE* gene pairs on chromosomes in intra-genomics of *G.arboreum, G. raimondii*, and *G. hirsutum*. The green boxes represent chromosomes and the red lines represent *CLE* homologous pairs.

**Figure 3:**
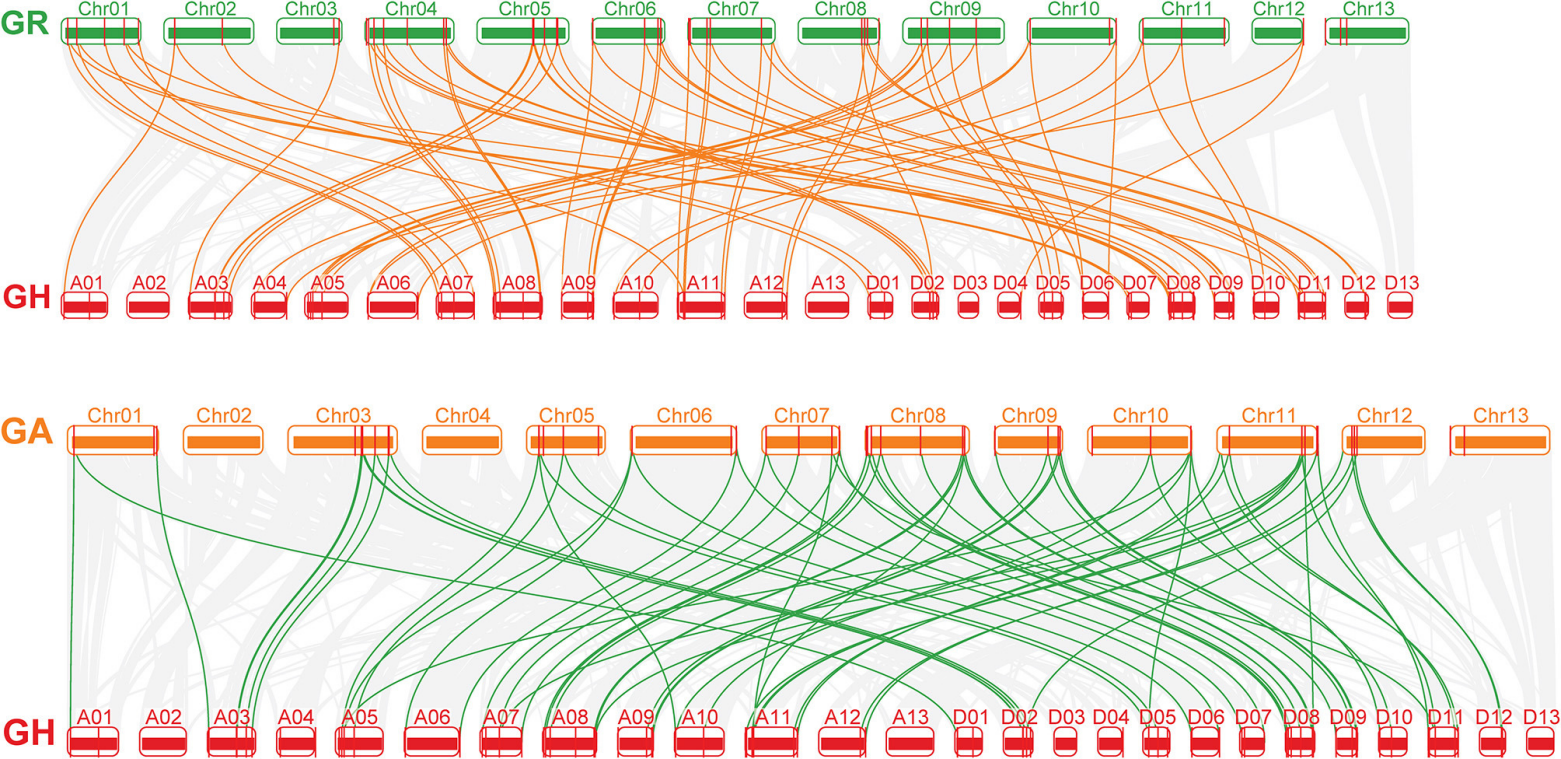
The collinear relationship between *G. arboreum* and *G. hirsutum* and between *G. raimondii* and *G. hirsutum* (from bottom to top). The gray lines: the collinearity of the whole genome among cotton. The yellow and green lines: the collinearity of *CLE* pairs in inter-genomics.

The collinearity of the *CLE* homologous genes in the three cotton genomes was analyzed. In total, 16, 21, and 46 pairs of *CLEs* intragenomic homologous genes were identified one by one in *G. raimondii, G. arboreum*, and *G.hirsutum* (Figure 2 and Supplementary Table 4). To understand the intergenomic collinearity, 73 and 38 collinear pairs of homologous genes were predicted between *G. hirsutum* and *G. arboreum/G. raimondii*, respectively. Interestingly, most of the *GaCLE* members corresponded to two *GhCLE* members, and one *GrCLE* member generally corresponds to only one *GhCLE* (Figure 3 and Supplementary Table 4). Furthermore, 39 pairs of genes derived from whole genome duplications or segmental duplications in *G.hirsutum*, and 40 or 47 pairs between *G. hirsutum* and *G. arboreum*/*G.raimondii* were identified. High-copy genes exist in most groups. The details of the type of duplication undergone by the collinear gene pairs are listed in Supplementary Tables 5–7.

The selection pressure for *CLE* gene duplication was assessed using the Ka/Ks ratio (Supplementary Tables 5–7). Usually, a Ka/Ks value <1, indicates purifying selection, Ka/Ks = 1 indicates neutral evolution, and Ka/Ks > 1 indicates directional selection (Mayrose et al., 2007). Most of the Ka/Ks values were <1, indicating that the *CLE* gene family in cotton had undergone purifying selection during the long evolutionary process. Nevertheless, several gene pairs (*GhCLE7.2/GhCLE7.1, GhCLE21.2/GhCLE21, GhCLE5.2/GaCLE5, GhCLE7.2/GaCLE7, GrCLE4/GhCLE4.1, and GrCLE18/GhCLE18.3*) had Ka/Ks values >1, indicating that they may have been formed according to directional selection.

### Analysis of *cis*-Acting Regulatory Elements of *GhCLE* Genes

**Figure 4:**
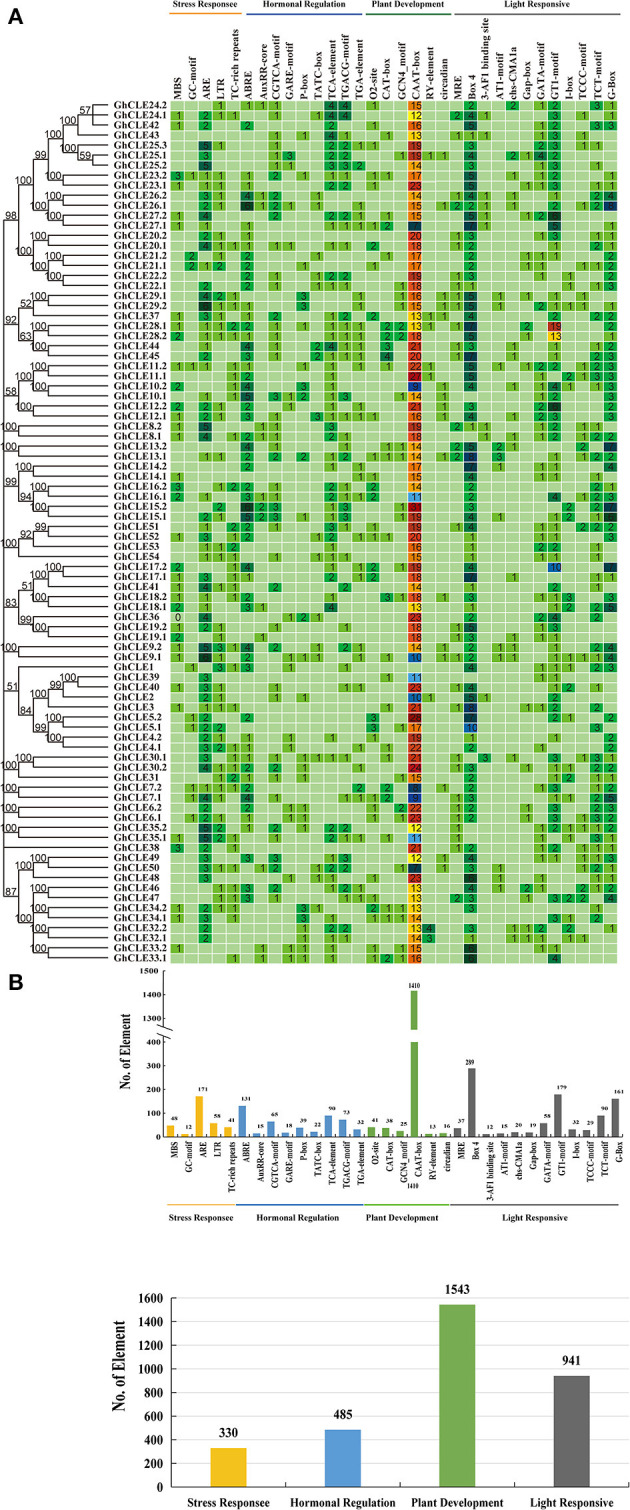
Analysis of *C*is-acting element numbers in *G. hirsutum* CLE peptide signals. **(A)** The colors of the grid indicate the number of *C*is-acting elements of the *GhCLE* genes. **(B)** The bar chart represents the sum of each type of *C*is-acting elements.

To identify the potential function of cotton *CLE* genes, 32 types of *cis*-acting regulatory elements (CAREs) were obtained from *GhCLE* members, and grouped into four functional types as shown in Figure 4A. (i) Involved in stress responses, such as MYB binding sites (MBS) that result in drought-inducibility, anaerobic induction regulatory elements (ARE), low-temperature responsive elements (LTR), defense and stress responsive elements (TC-rich repeats), and anoxic specific inducibility (ARE) etc.; (ii) involved in hormonal regulation, including gibberellin-responsiveness (GA) elements (GARE-motif, TATC-box, P-box), abscisic acid (ABA) responsive elements (ABRE), auxin-responsive (IAA) elements (TGA, AuxRR-core), salicylic acid (SA) responsiveness (TCA), MeJA-responsive elements, and (CGTCA-motif, TCACG-motif); (iii)involved in plant development (CAAT-box, CAT-box, etc.); and (iv) involved in light responsive elements (GT1-motif, Box4, G-Box, etc.).

Most CAREs were found to be involved in plant growth and development (all 1,543), especially the CAAT elements, accounting for 91% of them all. Almost all members contained 7 to 31 CAAT elements, except *GhCLE1*. The GCN4_motif is involved in endosperm expression, and the CAT-box element is related to meristem expression. Light responsiveness (941) and hormonal signaling (485) elements were the next to be identified. Nine and 19 types of hormonal signaling and light response- related CAREs were identified in *GhCLE* promoters, respectively. As for stress response elements (330), ARE was found to be the most abundant in the stress response group (accounting for 51%). More importantly, the CAREs, involved in drought-inducibility, only represented 14.5% of the total, while other stress-related *cis*-elements were identified and found to be involved in multiple abiotic stresses. More than one putative stress-related responsive element was identified in all *GhCLE* members (except *GhCLE43, GhCLE22.2, GhCLE13.2*, and *GhCLE14.2*) (Figure 4B).

In addition, highly similar types and quantities of CAREs among homologous genes were analyzed. Five pairs of ARE, involved in drought stress regulation (*GhCLE9.1/GhCLE9.2, GhCLE35.1/GhCLE35.2, GhCLE8.1/GhCLE8.2, GhCLE29.1/GhCLE29.2*, and *GhCLE25.1/GhCLE25.2/GhCLE25.3)* and *GhCLE28.1* and *GhCLE28*.2 contained 19 and 13 GT1-motifs, involved in light responses, respectively, were identified (Figure 4).

### *GhCLE* Expression Patterns Analysis in Specific Organs and Tissues

**Figure 5:**
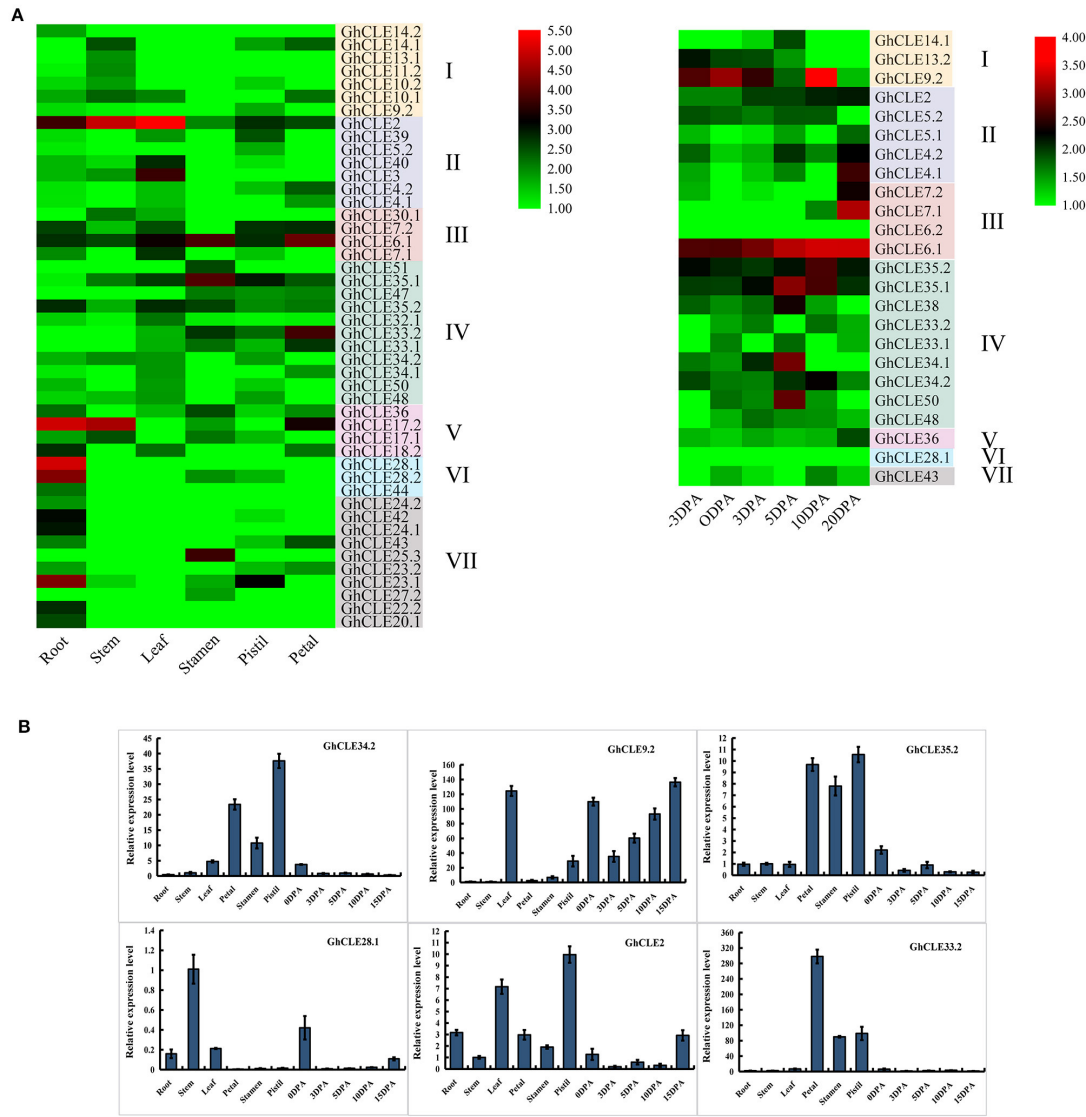
Expression patterns of *GhCLEs* in different tissues. **(A)** Tissue/organ-specific expression patterns of *GhCLEs*, including root, stem, leaf, petal, pistil, stamen, and ovule (−3, 0, 3, 5, 10, 20 DPA). Heat map was drawn according to calculation results of log_2_ of fragments per kilobase million (FPKM) RNA sequencing (RNA-seq) data. **(B)** Relative expression levels of six *GhCLE* genes in different tissues.

As important indicators of gene biological function, tissue- and organ-specific gene expression profiles were considered to be crucial. As shown in Figure 5A, *GhCLE* members were widely expressed in petals, stamens, pistils, and −3, 0, 3, 5, 10, and 20 days post-anthesis (DPA) ovules (reproductive organs) and in roots, stems, and leaves (vegetative organs). Many *CLEs* of Group III and IV (possess similar DPXHN motif in Figure 1A2) showed high expression levels in the reproductive tissues. For example, the homologous genes, *GhCLE33.2, GhCLE33.1*, and *GhCLE47* showed significant levels of expression in the petals, stamens, and pistils. *GhCLE6.1, GhCLE2*, and *GhCLE35.2* were highly expressed in all tested tissues. Most of the member*s* of Groups V, VI, and VII (possess similar PGGP motif in Figure 1A2) were specifically expressed in root, especially *GhCLE28.1, GhCLE28.2, GhCLE24.1*, and *GhCLE42*, and *GhCLE9.2* exhibited the highest expression level in 10 DPA ovules. Generally, the expression patterns of the *GhCLE* gene pairs showed high similarity. For example, *GhCLE35.1* and *GhCLE35.2* were significantly expressed in flower organs, leaves, and all stages of ovule development, while *GhCLE28.1* and *GhCLE28.2* were highly expressed in the roots. Nevertheless, the expression patterns of the two were not always the same between paralogous genes. For instance, *GhCLE17.2* was predominantly expressed in roots, stems, and petals but *GhCLE17.1* was not expressed at a low level in petals. *GhCLE23.1* was highly expressed in roots and pistils but *GhCLE23.2* was mainly expressed in roots and petals. *GhCLE6.1* was highly expressed during ovule development, while *GhCLE6.2* was not.

Based on the analysis of *G. hirsutum* expression profile, six genes (*GhCLE34.2, GhCLE9.2, GhCLE35.2, GhCLE28.1, GhCLE2*, and *GhCLE33.2*) were randomly selected for further RT-PCR verification. Four of six genes were highly expressed in the flower organs (petals, stamens, and pistils). *GhCLE34.2, GhCLE35.2*, and *GhCLE33.2* of Group IV were significantly expressed in the flower organs, while *GhCLE2* of Group II was mainly expressed in the pistil or leaves. *GhCLE9.2* of Group I was distinctly expressed in leaves and ovules, while *GhCLE28.1* of Group VI was prominently expressed in the stem (Figure 5B). The results of the qRT-PCR analyses were basically coincident with those obtained through RNA-seq (Figure 5).

### *GhCLE* Expression Pattern Analysis in Various Abiotic Stresses

**Figure 6:**
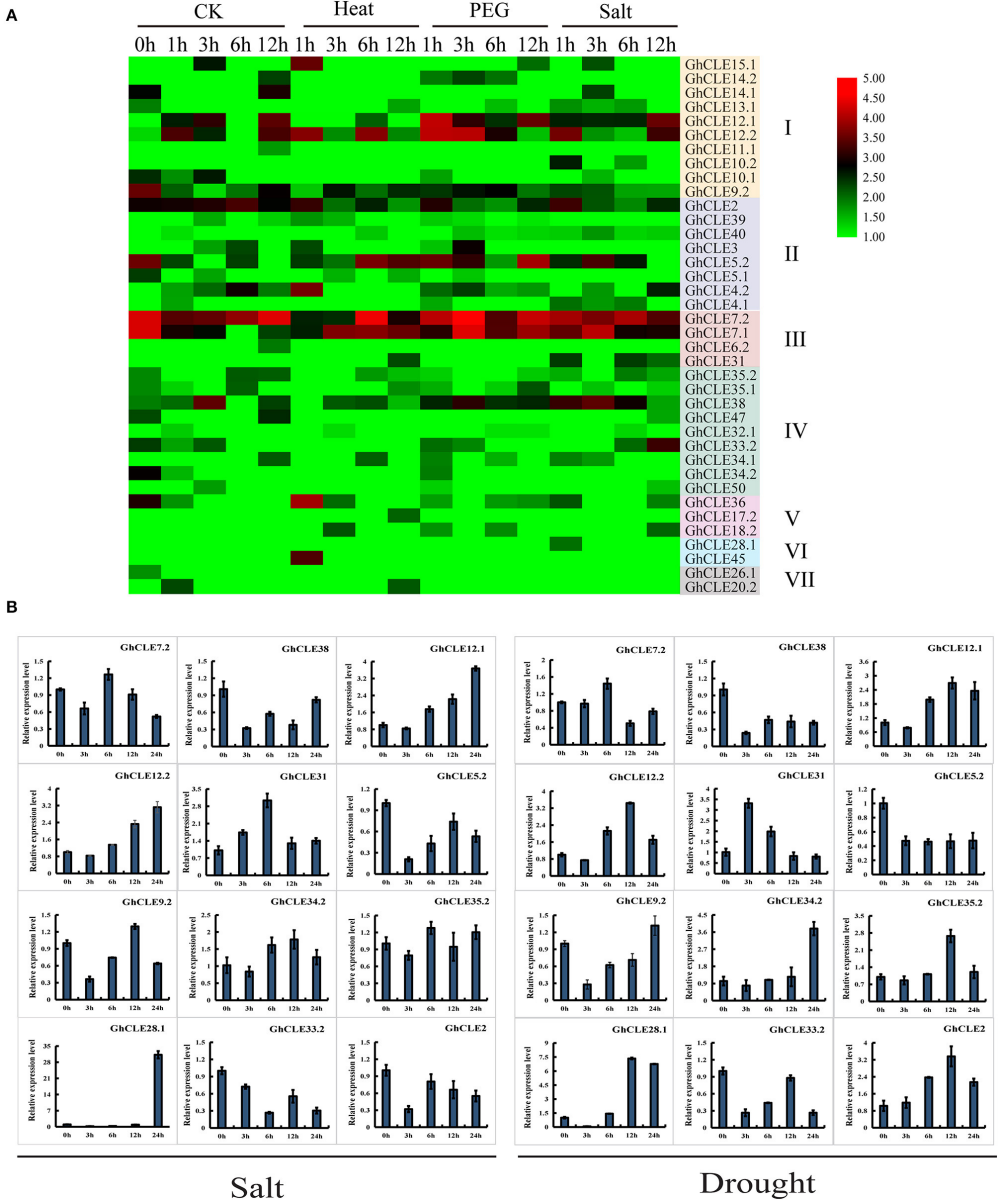
Expression patterns of *GhCLEs* under different stress. **(A)** Stress-induced expression patterns of *GhCLEs* under polyethylene glycol (PEG), and salt treatments for 1-, 3-, 6-,12-, and 24-h, respectively. Heat maps are drawn in the same way as in Figure 5. **(B)** Relative expression levels of 12 *GhCLE* genes under different stress.

In order to understand the putative functions of *GhCLE* genes, a comprehensive analysis of the expression patterns was carried out under heat, salt, and drought treatments (Figure 6A). Forty-eight out of the 86 *GhCLEs* were barely expressed at all time points (these are not shown in the heat map). When the cotton plants were exposed to three stress treatments, more than half of the members were less or almost imperceptibly expressed, especially those in Groups IV, V, VI, and VII. *GhCLE12.1* and *GhCLE12.2* of Group I were significantly expressed for 1 h of drought treatment, and a relatively high expression was observed at 12 h under salt treatment. Among the Group III genes, *GhCLE7.1* and *GhCLE7.2* were notably expressed at the 3 h time point of drought stress and modestly expressed at other time points compared with the other members. *GhCLE38* of Group IV was highly expressed at 1, 3, and 6 h of the salt and drought treatments, while *GhCLE15.1* of Group I and *GhCLE36* of Group V were only expressed at 1 h of heat treatment.

According to the heatmap, the expression of 6 genes was increased at certain periods under the three stresses. This finding was further verified by qRT-PCR analysis with three technical repetitions. Three *GhCLEs* (*GhCLE38, GhCLE5.2*, and *GhCLE33.2*) were constantly downregulated under salt and drought treatment. The expression levels of *GhCLE7.2, GhCLE31*, and *GhCLE35.2* were the highest at 6 h. Homologous genes, such as *GhCLE12.1* and *GhCLE12.2* showed continuous upward expression after salt stress and were predominantly expressed at 12 h after drought stress. *GhCLE9.2* and *GhCLE34*.2 were significantly expressed at 12 h after salt treatment and 24 h after drought treatment, while *GhCLE2* was continuously downregulated under salt treatment and showed the highest expression level at 12 h after drought stress. *GhCLE35.2* and *GhCLE28*.1 were also highly expressed at 12 h after drought stress. Members of the same subfamily showed not only functional redundancy but also functional differentiation. For instance, *GhCLE12.1, GhCLE12.2*, and *GhCLE9.2* in Group I were upregulated under salt and drought treatment. The expression levels of *GhCLE34.2* and *GhCLE35.2* in Group IV were upregulated under the two stresses, while *GhCLE33.2* and *GhCLE38* were downregulated (Figure 6B).

### Analysis of Physical and Chemical Properties, Structure Prediction and Interaction Network of GhCLE Proteins

**Figure 7:**
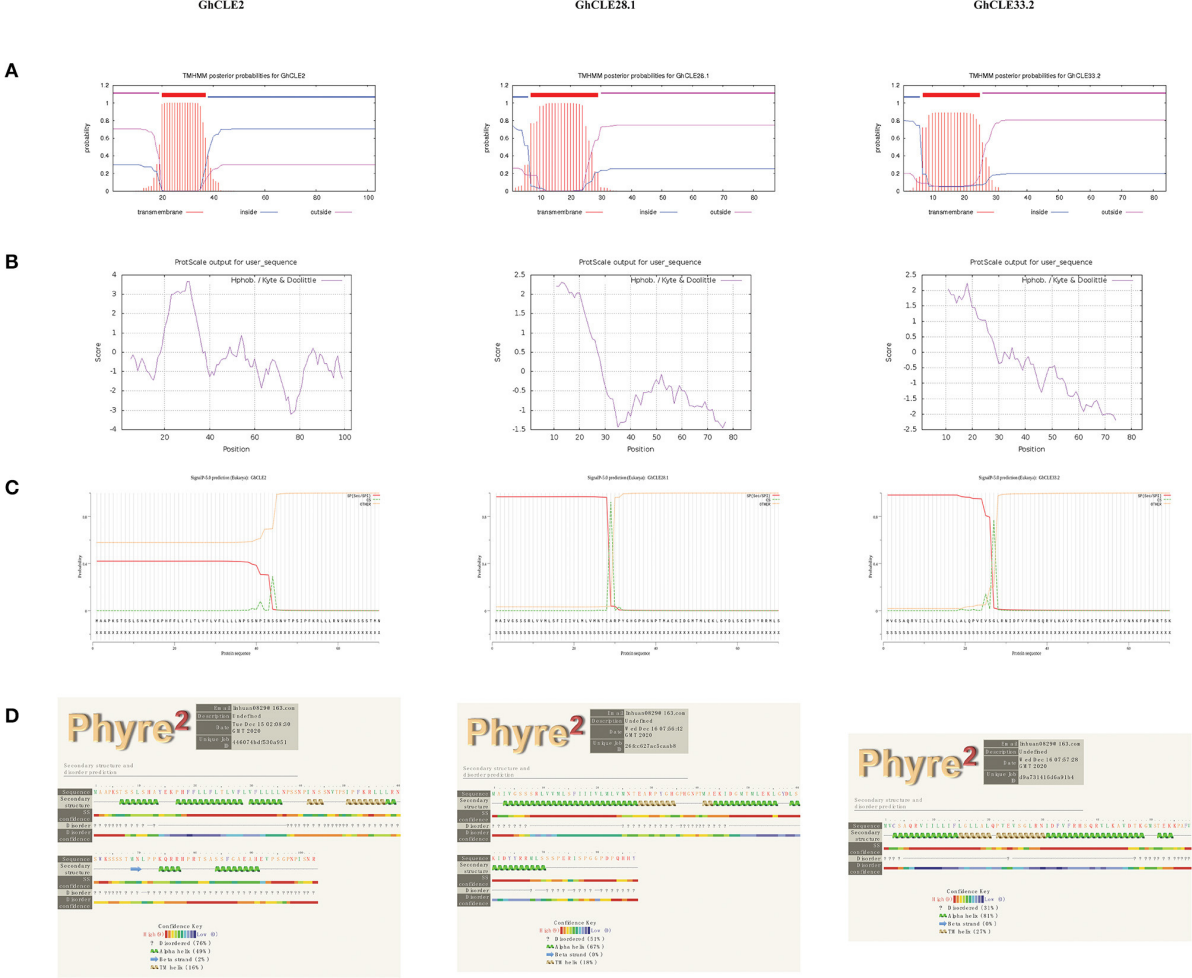
**(A–D)** Respectively represent transmembrane structure characteristics, hydrophobicity/hydrophilicity, signal peptides, and secondary structure of GhCLE2, GhCLE28.1, and GhCLE33.2.

To understand the relationship between the structure and function of CLE proteins, GhCLE2, GhCLE33.2, and GhCLE28.1 peptides from different groups were analyzed. The three GhCLEs belonging to membrane proteins were found to form transmembrane region (close to N-terminal), extracellular region, and intracellular region (some proteins have one or more transmembrane regions) (Figure 7A). The number of hydrophilic amino acid residues was more than that for hydrophobic residues (a positive value represents hydrophobic residues and a negative value indicates hydrophilic residues) (Figure 7B). The signal peptide regions were analyzed at the N-terminals of three protein sequences, but GhCLE2 was less likely to have a signal peptide (42.21%) (Figure 7C). The secondary structures of the proteins were predicted as follows: theα-helix was dominant in the polypeptide chains of GhCLE28.1 and GhCLE33.2, followed by disordered structures and TM helices. The GhCLE2 protein contained 76% disordered structures, 49% α-helices, 16% TM helices, and 2% β-turn (Figure 7D).

**Figure 8:**
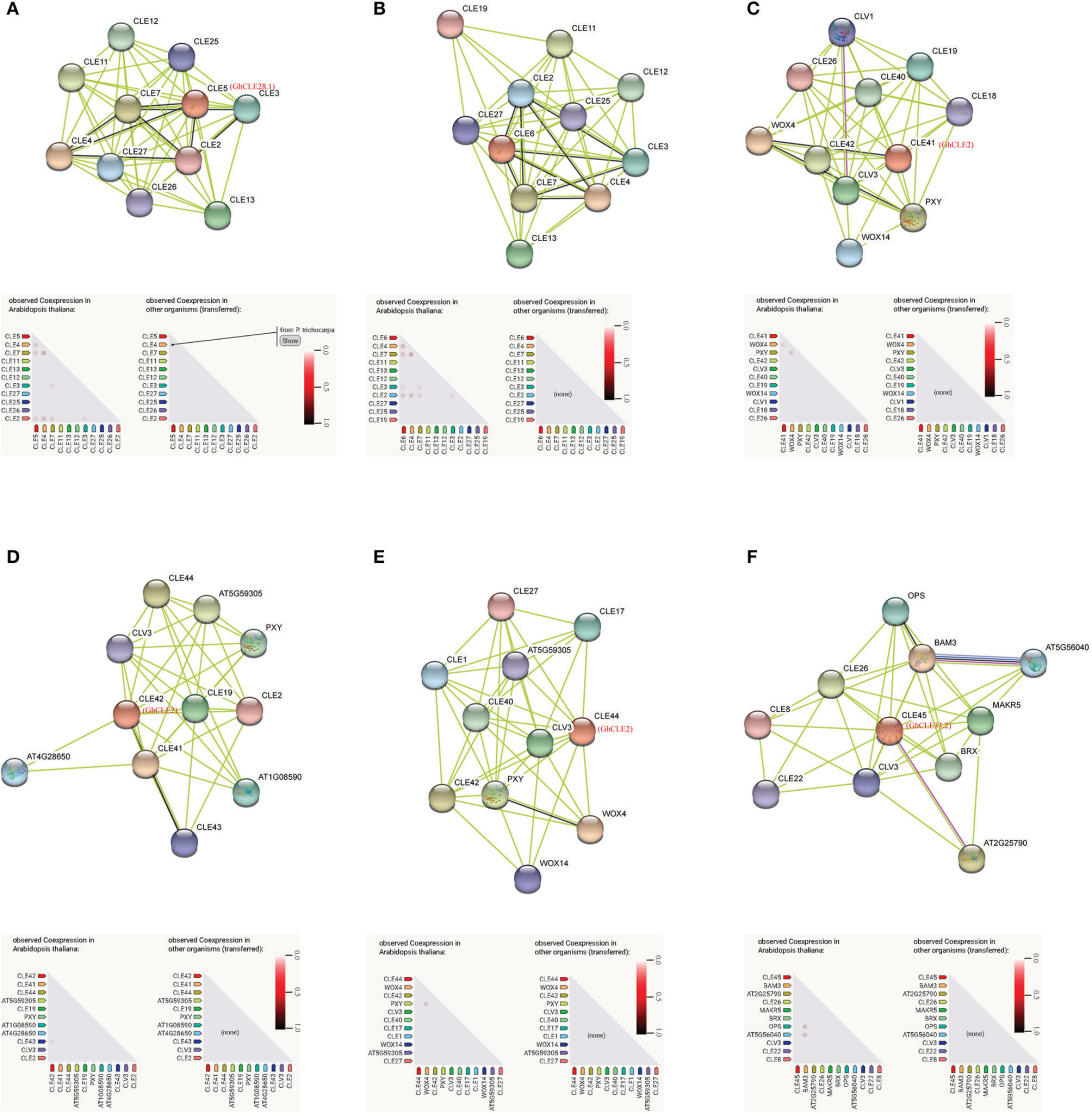
Prediction of interaction network between CLE signal peptide and other related proteins in *G. hirsutum*, with homologous protein in *Arabidopsis* as enquiry protein. Homologous proteins of *Arabidopsis* in *G. hirsutum* are shown in red. The black line represents the co-expressive relationship. The co-expression predicts functional association under the network diagram: in the triangle matrix, the intensity of the color indicates the level of confidence that two proteins are functionally associated. **(A)** CLE5; **(B)** CLE6; **(C)** CLE41; **(D)** CLE42; **(E)** CLE44; **(F)** CLE45.

Based on the findings in *Arabidopsis*, the interactions and regulatory pathways of homologous CLE peptides were predicted in cotton. There were three CLEs (GhCLE28.1, GhCLE2, and GhCLE33.2) in *G. hirsutum* corresponding to six genes in *Arabidopsis*. Ten high evident interactive proteins and co-expression proteins in *Arabidopsis* and other organisms were investigated in every query protein (Figure 8 and Supplementary Table 8). CLE5 and CLE6 were co-expressed with four genes (CLE2, CLE3, CLE4, and CLE7) within the family, respectively. They were closely related to GhCLE37, GhCLE28.1, and GhCLE28.2. While CLE41 (GhCLE2) co-expressed only with WOX4, CLE42 (GhCLE2) co-expressed with CLE43; even CLE44 (GhCLE2) and CLE45 (GhCLE33.2) had no co-expressed genes. Interestingly, CLE4 and CLE5 were co-expressed in *Populus* (Figure 8A). WOX4 and WOX14 played a role downstream of the PXY receptor kinase in the GhCLE2 (CLE41, CLE42, and CLE44) network (Figures 8C–E). Moreover, the interactive proteins involved in the GhCLE33.2 network included some receptor-like kinases (LRR-RLKs), such as BAM3, MAKR5, BRX, OPS, CLV3, CLE8, CLE22, CLE26, OPS, AT5G56040 (RGF1), and AT2G25790 (SKM1) (Figure 8F).

### Overexpression Analysis of *GhCLE2, GhCLE33.2*, and *GhCLE28.1* in *Arabidopsis*

**Figure 9:**
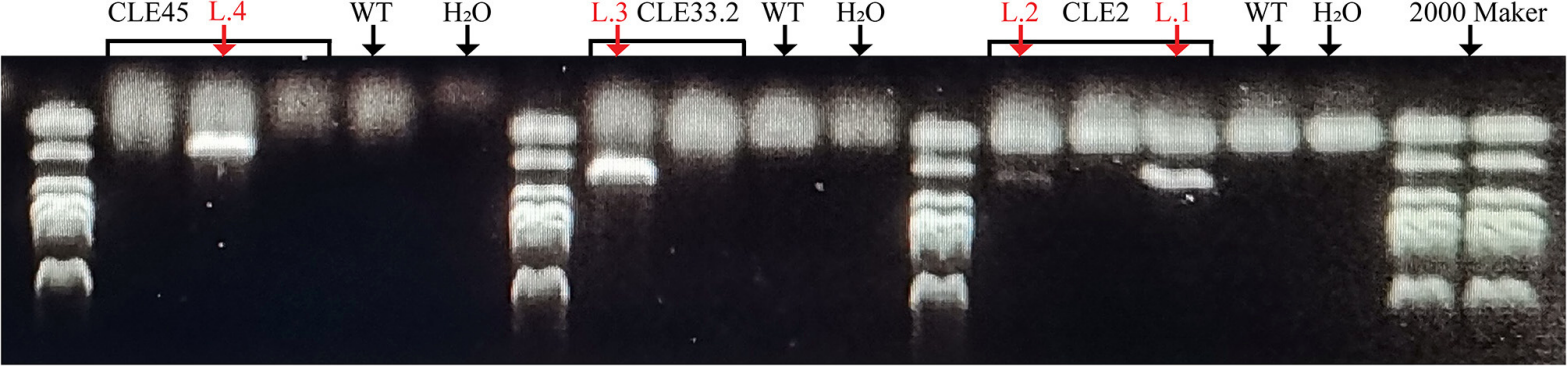
The transgenic lines were verified by Agarose gel electrophoresis. The red arrows point to successful transgenic lines.

**Figure 10:**
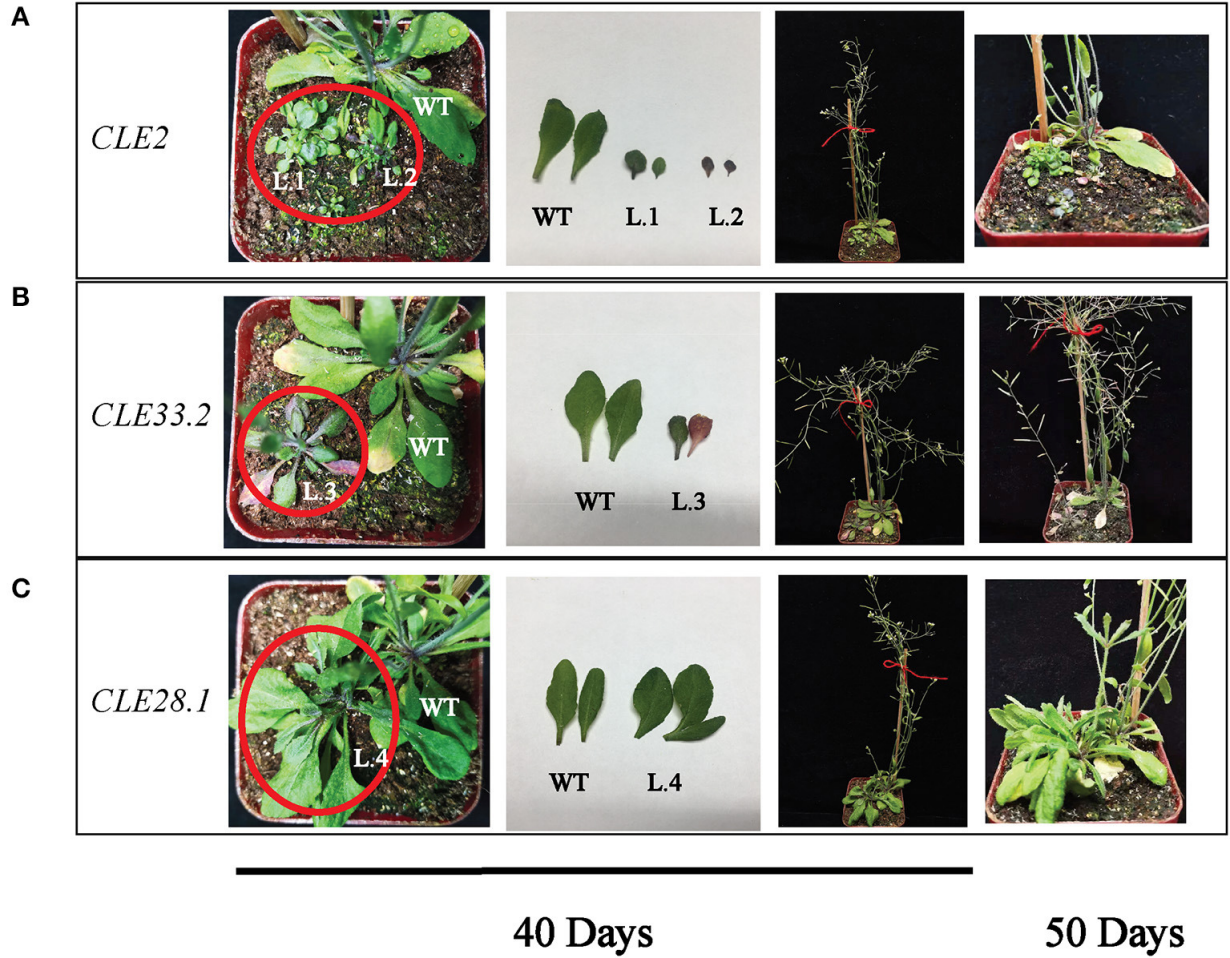
Phenotypes of *GhCLE2* **(A)**, *GhCLE33.2* **(B)**, and *GhCLE28.1* **(C)** overexpressed in *Arabidopsis*. The pictures of the first three columns show the seedlings and corresponding leaves at 40 days after germination, and the last column shows seedlings at 50 days old.

To clarify the biological functions of *CLEs* in cotton, three *GhCLE* genes were overexpressed in *Arabidopsis*. *GhCLE2* was widely expressed in the most tested tissues, especially in leaves and stems; *GhCLE33.2* was highly expressed in flower organs, notably petals; *GhCLE28.1* was specifically expressed in roots. The successful transgenic lines were screened out in Figure 9. Compared with the wild-type (WT), the *GhCLE2OE1* (L.1) and *GhCLE2OE2* (L.2) lines grew into dense dwarf plants and produced a large number of small leaves and formed small rosettes, exhibiting a shrub-like phenotype. These lines also expressed short stems and small siliques (Figure 10A). Overexpression of the *GhCLE33.2* gene in *Arabidopsis* was found to result in anthocyanin overproduction, especially in several old leaves and stems. The old leaves were light purple or even pink, while young leaves and stems were dark green or slightly purple. Moreover, line (L.3) showed mild developmental timing delays and formed small rosettes and dwarf phenotypes (Figure 10B). Unlike *GhCLE2OE* and *GhCLE33.2OE, GhCLE28.1OE* lines (L.4) displayed larger rosettes with leaves of misshapen character and abnormal enlargement, which also apparently result in short, flat-shaped stem with infertile flowers. In summary, the transgenic plants exhibited a significant decrease in plant height compared to the WT plants (Figure 10C). The above phenotypes of the overexpressed lines confirmed that the *CLE* family mainly regulates meristem and plays an important role in the growth and development of plants.

## Discussion

Over the past few years, CLE signal peptides have attracted attention from the scientific community. The biological function of peptides involved in plant growth, development, and responses to environmental stresses have become more evident, with a large number of peptide family members identified. Previous genome-wide studies of the *CLE* family have focused mainly on *Arabidopsis, Oryza sativa, Glycine max, Zea*, and *Triticum aestivum* (Opsahl-Ferstad et al., 1997; Suzaki et al., 2006; Oelkers et al., 2008; Hastwell et al., 2015a; Li et al., 2019). However, the completion of the cotton whole-genome sequencing project provides a chance to characterize the *CLE* gene family in this genus. Comprehensive identification of CLE peptides was performed in three cotton species. Eighty-six *CLE* members were identified in *G. hirsutum*, 56 in *G. arboreum*, and 55 in *G. ramondii*. Subsequently, a series of evolutionary analyses were performed, including chromosomal location, gene structure, identification of conservative domains, collinearity, protein properties and interaction, expression patterns, and functional verification.

Although the diploids, *G. arboreum* and *G. ramondii* are the ancestors of *G. hirsutum*, the distribution of *CLE* genes on their chromosomes are not greatly regular (Zhang et al., 2015). *G. arboreum*, the A-genome donor of *G. hirsutum*, has no CLE genes on chromosome Ga02 and Ga04. It is also found that there are no CLE genes on chromosome A13 of *G. hirsutum* while there are two genes on A04. In *G. ramondii*, the D-genome donor of *G. hirsutum*, all genes are irregularly distributed on all the 13 chromosomes, while there is no member on chromosomes, D03 and D13 of *G. hirsutum*. The number of *CLE* family genes in *G. hirsutum* theoretically should have been approximately twice that in diploid cotton, but it was found only to be 1.5-fold. Many of the *CLE* genes in *G. hirsutum* were considered double genes due to the synteny of the A and D subgenomes. Nevertheless, there were some exceptions that lacked one corresponding homologous gene. These counterparts may have been lost or became pseudogenes in their repeated regions during evolution process of *G. hirsutum* (Magadum et al., 2013; Iranzo et al., 2019). In addition, the number of cotton *CLE* genes was considerably more than that seen presently in grape (nine members) (Wang et al., 2019), *Arabidopsis* (at least 32) (Jun et al., 2010), common bean (44) (Hastwell et al., 2015a) and rice (47) (Suzaki et al., 2006; Kinoshita et al., 2007), but less than in hexaploid bread wheat (*Tricticumaestivum L*.) (104) (Li et al., 2019). However, the number of *CLE* genes (50 members) in *Populus* was similar to those in the A (*G. arboreum*) or D (*G. ramondii*) genomes (Liu et al., 2016). The quantity of *CLE* members in the allotetraploid species (*G. hirsutum*) was very close to that in soybean (84 *CLEs*) (Hastwell et al., 2015a). Apparently, if the genome size between species is significantly different, the number of *CLEs* is also different.

Segmental duplications appear most frequently in polyploid plants and reserve numerous duplicated chromosomal blocks within their genomes, such as diploid *Arabidopsis* (Cannon et al., 2004). In this study, 46 pairs of whole-genome or segmental duplications among 86 *GhCLE* genes, 73 pairs between *G. hirsutum* and *G. arboreum*, and 38 between *G. hirsutum* and *G. raimondii* were identified. Therefore, whole-genome or segmental duplications might have been one of the primary driving forces for *CLE* family expansion during the evolution of cotton, likely with SPL transcription factors (Cai et al., 2018). Group VII contained the most *GhCLE* members, including whole-genome or segmental duplications, thus introducing functional redundancy during the regulation of cotton development and responses to abiotic stress. However, the main expansion approach of the *CLE*s in wheat was tandem duplication (Li et al., 2019). Although wheat is of the same gene family, the way of amplification is probably different among species. These results may facilitate the characterization of new biological functions of new *CLE* genes (Cannon et al., 2004). Interestingly, previous studies found that the MW of all *CLE* family genes in *Arabidopsis* is <15 kDa, while 16 cotton CLE peptides are >15 kDa. In particular, four genes encode proteins >20 kDa (GhCLE51/GhCLE52/GhCLE53/GhCLE54 belonging to Group IV) in *G. hirsutum* (Cock and McCormick, 2001). These genes were speculated to be involved in C-terminal extension, as similar genes, such as *AtCLV3, ZmESR3, PtCLE1*9, and OsCLE32, have been identified (Kinoshita et al., 2007; Oelkers et al., 2008; Wang and Fiers, 2010; Han et al., 2016). The proteins containing more than 200 amino acids along their length were located in the nucleus and had multiple CLE domains. *GhCLE51/GhCLE54 and GhCLE52/GhCLE54* were two pairs of homologous genes located on the chromosomes, GhA11 and GhD11, respectively. These features indicated that they arose by segmental or whole-genome duplication and possess a quite close relationship. Therefore, their functions were thought to be redundant and different from other members but these are unknown.

Seven gene clusters were identified in cotton species. It was beneficial to elucidate similarities in the function of the latest *CLE* members and their related homologs and orthologs (Han et al., 2016; Hastwell et al., 2017b). Different numbers of gene clusters were found in different species in previous studies: three, four, five, and seven gene clusters were identified in grape, populus, wheat, and soybean, respectively (Hastwell et al., 2015a; Liu et al., 2016; Li et al., 2019; Wang et al., 2019). Residues of the *CLE*-conserved domain have undergone diverse changes during the evolution of species, resulting in different classifications and diverse functions. The CLE motif of Group I had the highest sequence similarity to the CLV3 motif (LRTVPSGPDPLHH), functioning in shoot and root meristems (Kondo et al., 2006). Group II showed the highest similarity to the TDIF motif (HEVPSGPNPISN), which acts in vasculature (Etchells et al., 2016). The CLE motif of the other groups was very diverse and showed significant differences to the motifs of the seven groups in soybean (Hastwell et al., 2015a). The biological functions of some cotton *CLE* genes that diversified during evolution are unknown. Therefore, whether the variation in residues of different groups contributed to functional differentiation needs to be further predicted and verified. *Arabidopsis* CLE peptides can be divided into two types, A-type and B-type, according to their biological functions. A-types affect cell differentiation in root and shoot apical meristem (*AtCLE1, AtCLE4, AtCLE7, AtCLE19, AtCLE22*, and *AtCLE40*) (Whitford et al., 2008; Yamaguchi et al., 2016), and B-types affect vascular development (*AtCLE41-AtCLE44/TDIF*) (Strabala et al., 2006; Oelkers et al., 2008; Whitford et al., 2008; Qiang et al., 2013). To determine the functional classification of cotton CLE peptides, the members of *G. hirsutum* and *Populus* were together grouped into seven gene clusters. In this study, nine *GhCLE* genes belonged to Group II which contained four *AtCLE*s (*AtCLE41/AtCLE44/AtCLE42/AtCLE46*) and nine *PtCLEs*. These *CLE* genes classified together with *AtCLE41/TDIF*, have the same CLE motif, especially *GhCLE3*, which suggests that *GhCLE3* may affect vascular development (Whitford et al., 2008; Qiang et al., 2013).

An intriguing finding showed that some A-type *CLE* genes have distinctly specific expression patterns at certain development stages in the same tissues. *CLE3, CLE5, CLE16*, and *CLE17* were differently expressed in the primary or lateral roots of not fully mature and fully differentiated plants (Jun et al., 2010). Certain members (such as *GhCLE17.2, GhCLE28.1, GhCLE28.2*, and *GhCLE23.1*) of Groups V and VII may have similar functions such as the above genes. Previous studies have indicated that the *CLE5* and *CLE6* peptides singly and together showed the rosette leaf shape phenotype. These two genes are also expressed at the base above the abscission zone of the flower organ (Jun et al., 2010; DiGennaro et al., 2018). *GhCLE28.1* was found to be in the same subfamily as *AtCLE5* and *AtCLE6*. Overexpression of *GhCLE28.1* in *Arabidopsis* also shows enlarged rosettes and misshapen leaves, with a lack of apical meristem development. More importantly, the line did not form an inflorescence, so there was no progeny. The *GhCLE28.1* was also mainly expressed in stems, 0DPA ovules, leaves, and roots. The *GhCLE2* was widely expressed in most tested tissues, especially leaves, stems, and pistils. Overexpression of *GhCLE2* in *Arabidopsis* mainly exhibited distinctive shrub-like dwarf plants lacking apical dominance, suggesting that the proliferation and differentiation of meristems may be inhibited, including root, stem, flower, and meristem, which were akin to the phenotypes of overexpressing *AtCLE41, AtCLE42*, and *AtCLE44* (Strabala et al., 2006; Whitford et al., 2008). In this study, *GhCLE2* not only changed the size and number of the leaves but also affected the accumulation of anthocyanin (L.2). Uniquely, *GhCLE33.2OE* showed that the overproduction of anthocyanin is significant; the rosettes were also small but bigger than those of the *GhCLE2OE* lines; bolting occurred later compared to the WT lines, and a handful of seeds were harvested. Similar to the effect of most CLE peptides, *GhCLE33.2* also caused the entire line to be pygmean. Depuydt et al. (2013) showed that *AtCLE45* and the specific receptor *BAM3* affect proliferation and differentiation of protophloem, thereby influencing root meristem. The orthologous *GhCLE33.2* and *AtCLE45* have the same CLE motif (KRRVRRGSDPIHN) but the full-length protein sequence of CLE45 was longer than that of CLE33.2. However, *GhCLE33.2* was hardly expressed in roots, suggesting that serious functional differentiation may have occurred in this member. To sum up, the overexpression of the three *GhCLE*s in this study showed the same or similar phenotypes to overexpression lines of certain *CLEs* in *Arabidopsis* or other species, such as *AtCLE42, AtCLE44*, and wheat *TaCLE3* overexpression in *Arabidopsis* (Strabala et al., 2006; Li et al., 2019). To date, CLE peptides are proven to be involved in regulating stem cell homeostasis of the SAM and RAM, and they also regulate inflorescence development, lateral root growth, floral meristem formation, seed development and vascular formation (Clark et al., 1993; Strabala et al., 2006; Czyzewicz et al., 2013; Ingram and Gutierrez-Marcos, 2015). *G. hirsutum* serves as an ideal plant for different biological studies, such as genome evolution, polyploidization, and single-celled biological processes (Yin et al., 2021). Although some functions of *GhCLE* genes have been investigated, the more *CLEs* and their function need to be explored in the further.

The CLE peptide signaling pathway, which binds to phytohormone signaling pathways and perceives leucine-rich repeat receptor-like kinases (LRR-RLKs), is involved in the regulation of a variety of biological processes in response to environmental signals. As receptors, LRR-RLKs are perceived by CLE peptides, establishing the evolutionarily conserved CLE-RLK module, which can regulate plant development and responses to abiotic stresses by transmitting extracellular and intracellular signaling cascades (Betsuyaku et al., 2011; Murphy et al., 2012). This result was confirmed by the protein interaction analysis in this study. For instance, CLE (CLV1-like LRR-RLK) peptides play an eye-catching role in perceiving nitrate and controlling nodulation formation in legumes (Nishimura et al., 2002; Miyazawa et al., 2010; Miyawaki et al., 2013). Barely any meristem 3 (BAM3) acts as a receptor of CLE45 peptides to inhibit protophloem formation in *Arabidopsis* root meristems (Kang and Hardtke, 2016). In addition, *AtCLE25* peptides convey water-deficiency signals *via* the vascular tissues of *Arabidopsis* and work with BAM receptors in leaves to influence abscisic acid synthesis and stomatal transpiration control (Takahashi et al., 2018). According to evolutionary analysis, the GhCLE38-BAM module is most likely to act in long-distance signal transmission as a conveyer during the dehydration response. Therefore, the evolutionary characteristic and biological functions of the specific receptors of CLE ligands have gradually been identified and studied, providing guidance for further research on the function of cotton *CLE* genes.

In summary, *CLE* members in *G. raimondii, G. arboreum*, and *G. hirsutum* were identified, analyzed using bioinformatics, and their expression patterns and functions verified. The present study may provide insight into their structures and enable further investigation into the functions of cotton *CLEs* according to previously known orthologs in model plants and published transcriptional evidence. In order to further understand how certain key peptides regulate plant development and responses to environmental stimuli, the functional domain and structural modifications of these mature peptides need to be determined. Since cotton is a heterotetraploid species with a large genome, there will have been a variety of changes during evolution. Even if there are many functionally redundant members in a gene family, some genes will still be functionally differentiated. Therefore, finding and mining these genes to improve plant or crop characteristics is a scientific mission.

## Data Availability Statement

The original contributions presented in the study are included in the article/Supplementary Material, further inquiries can be directed to the corresponding authors.

## Author Contributions

HL, ZY, and WY conceived the research and designed the experiments. HL and WW conceived the structure of the manuscript and analyzed data and experiment results. HL wrote the initial manuscript. XC, XH, and SW participated in the experiments and modified the initial manuscript. ZS and YL participated in the second revision of the manuscript. All authors read and approved the final manuscript.

## Conflict of Interest

The authors declare that the research was conducted in the absence of any commercial or financial relationships that could be construed as a potential conflict of interest.
