# Supplementary material for: Molecular Traits and Functional Analysis of the CLAVATA3/Endosperm Surrounding Region-Related Small Signaling Peptides in Three Species of Gossypium Genus
Source: Front Plant Sci. 2021 Jun 4;12:671626. doi: 10.3389/fpls.2021.671626 (PMC8213210; doi:10.3389/fpls.2021.671626)
Supplement: Supplementary file 1 [file Data_Sheet_1.PDF]

*Supplementary Material*

# Supplementary Figures

**A.**

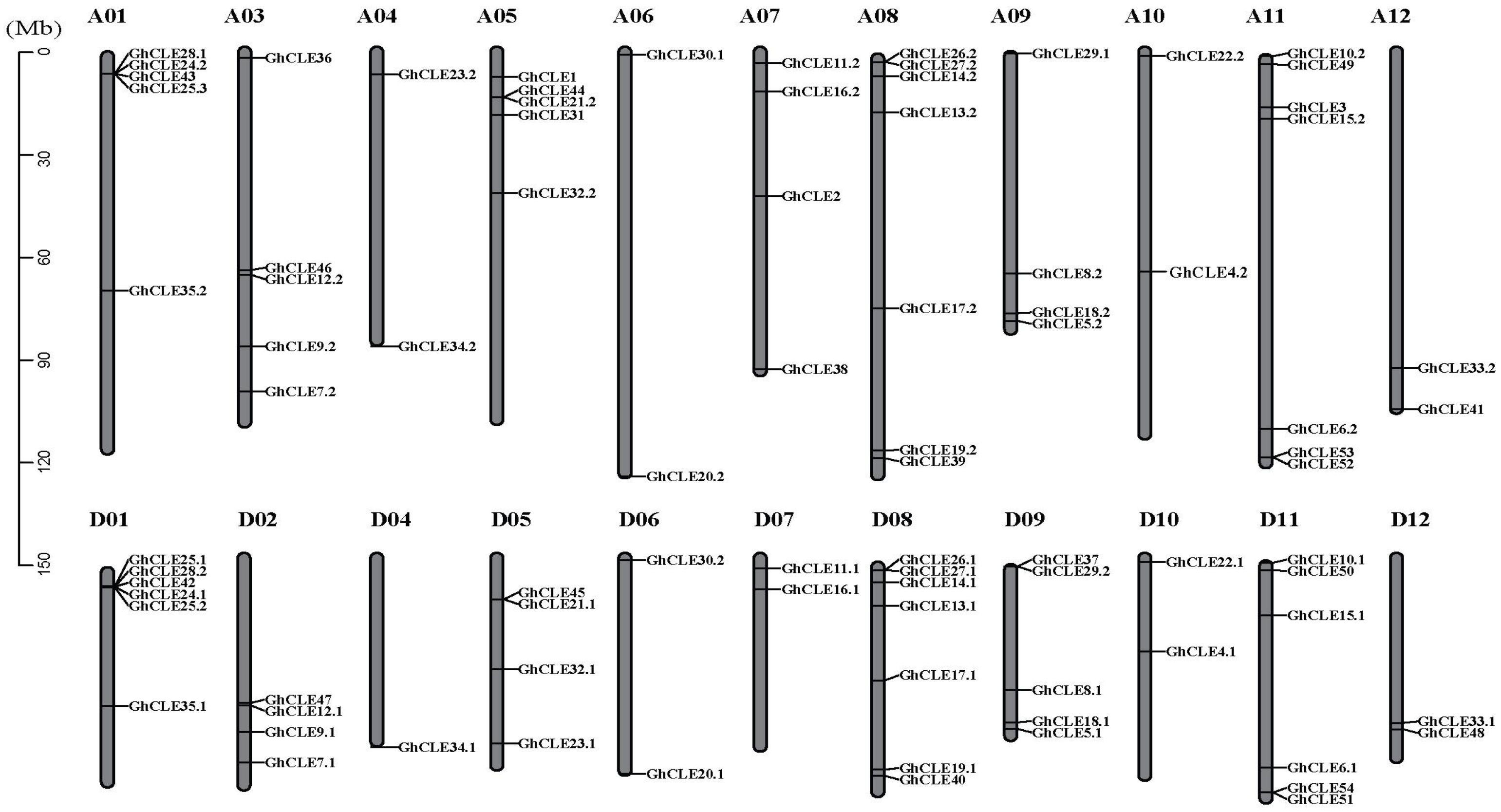

**B.**

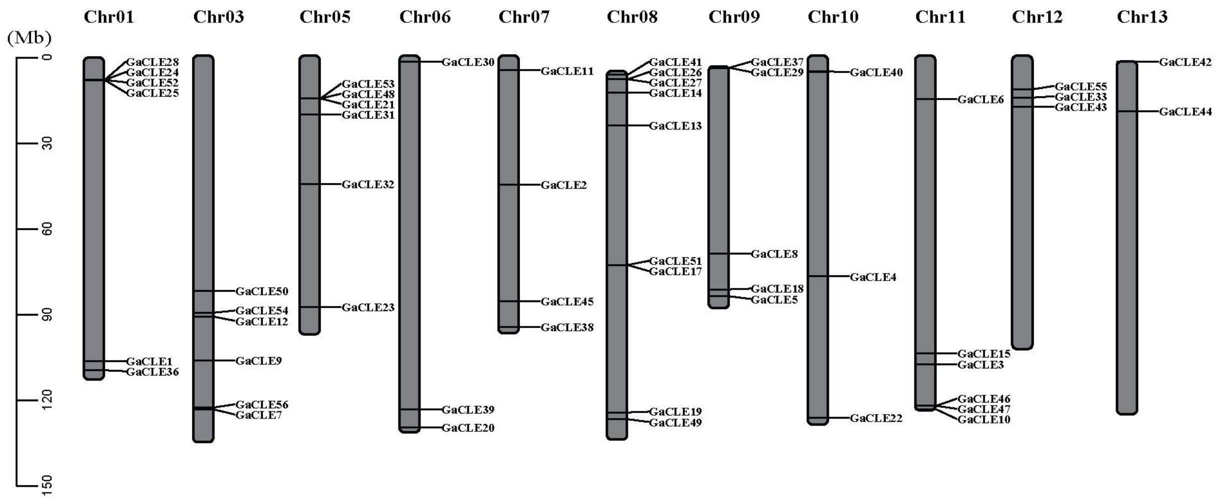


**C.**

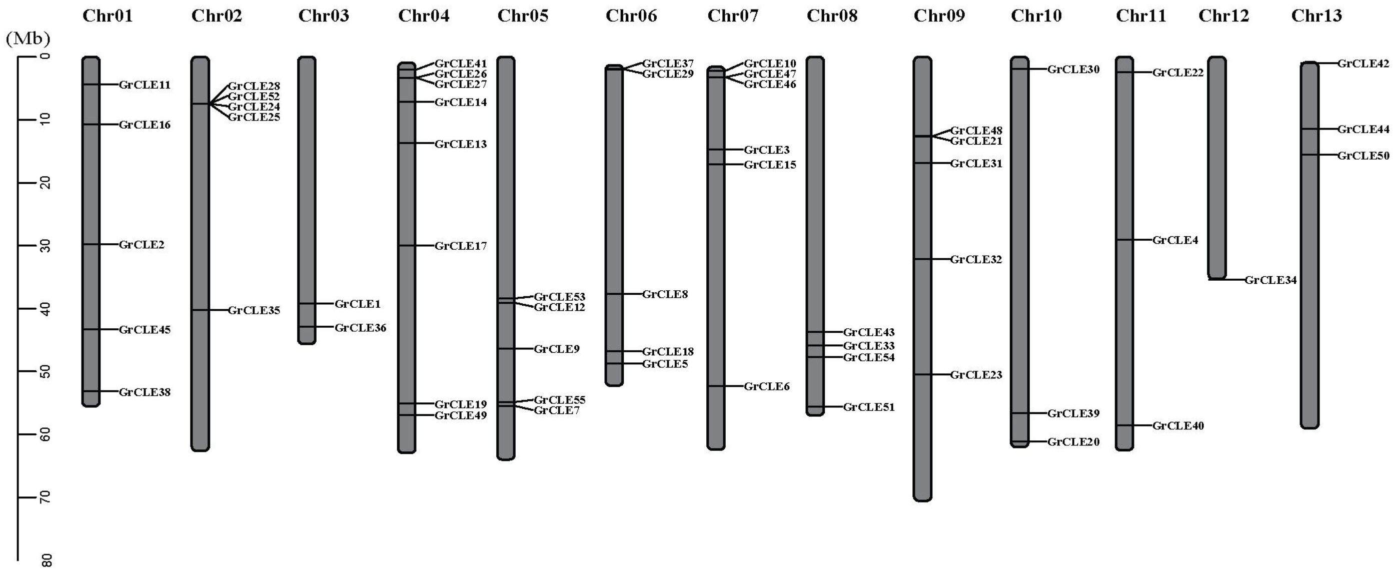


**Supplementary Figure 1**. A, B and C represent the chromosomal location of CLE gene on the chromosomes of *G.hirsutum*, *G.arboreum*, and *G. raimondii* , respectively. Above each gray bar are the names of chromosomes. The black line indicates the location of the corresponding gene. The vertical scale on the left indicates the size of the chromosome, and the scale represents the Mega Base (MB). The gene names correspond to Table 1-3.

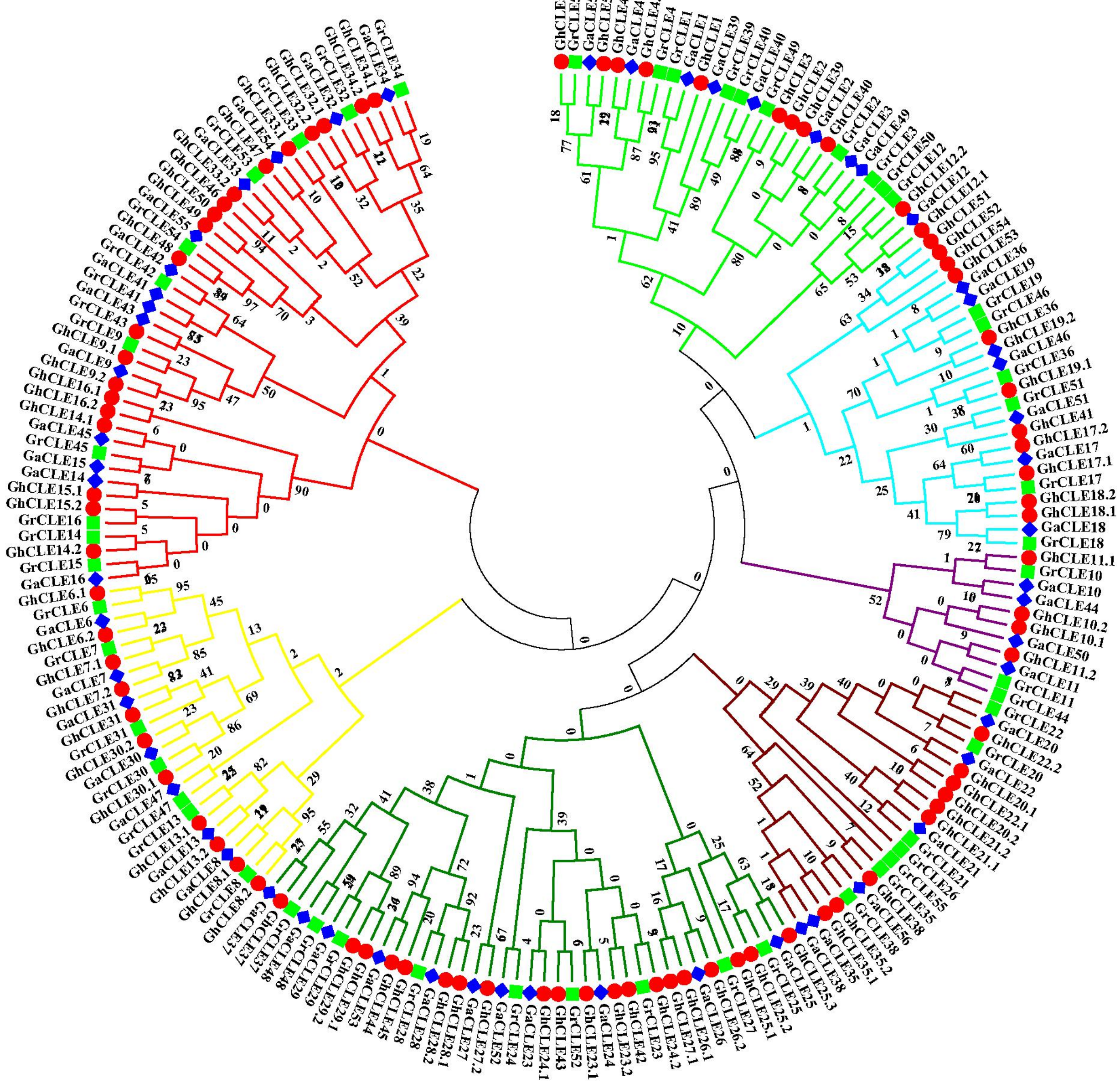

**Supplementary Figure 2.** Phylogenetic trees of *CLEs* among *G.raimondii*, *G.arboreum*, *and G.hirsutum* were established using MEGA7 with the ML (Maximum likelihood) method.

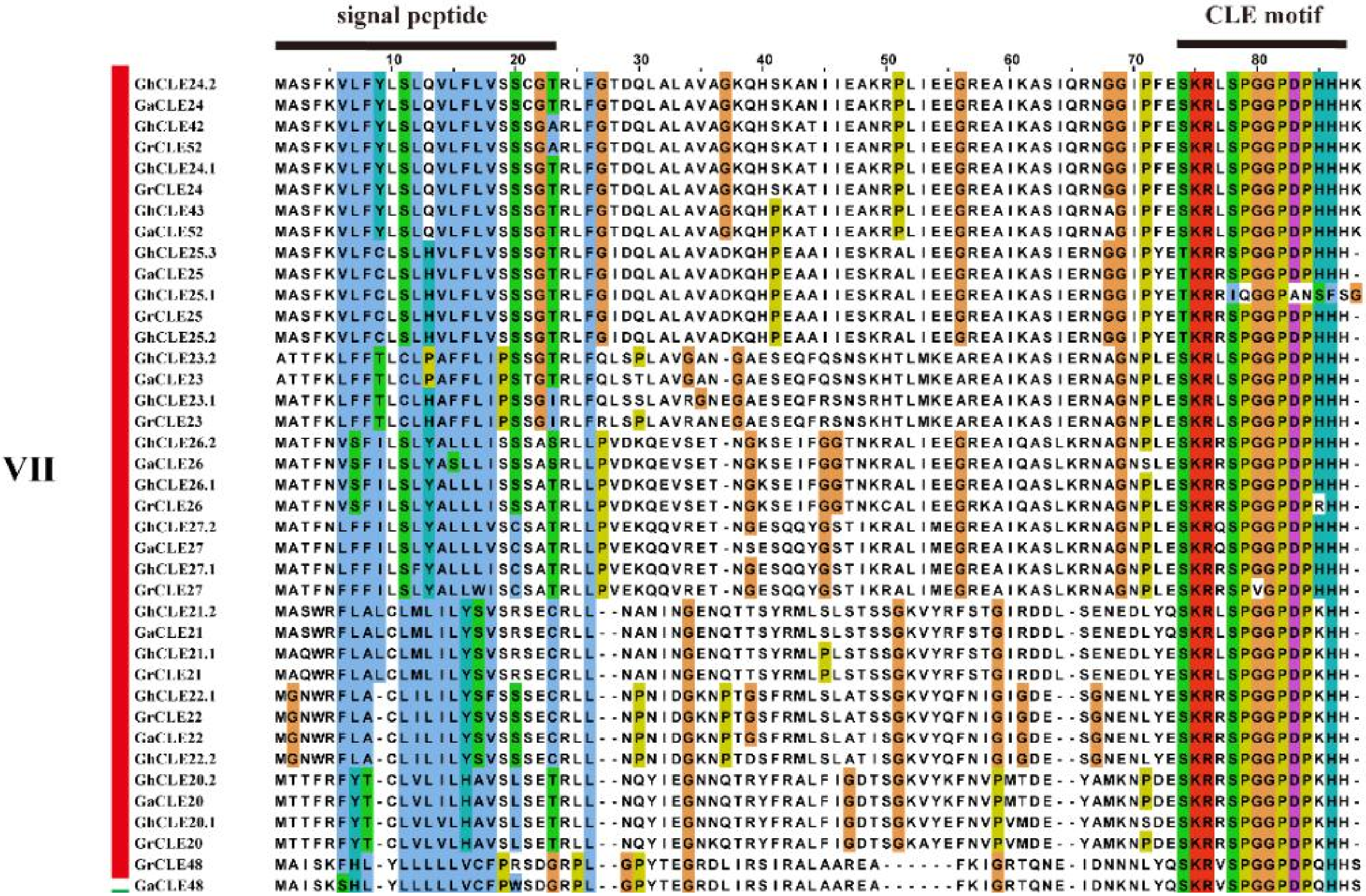
signal peptide
CLE motif
10
20
30
40
50
60
70
80
VII
GhCLE24.2
GaCLE24
GhCLE42
GrCLE52
GhCLE24.1
GrCLE24
GhCLE43
GaCLE52
GhCLE25.3
GaCLE25
GhCLE25.1
GrCLE25
GhCLE25.2
GhCLE23.2
GaCLE23
GhCLE23.1
GrCLE23
GhCLE26.2
GaCLE26
GhCLE26.1
GrCLE26
GhCLE27.2
GaCLE27
GhCLE27.1
GrCLE27
GhCLE21.2
GaCLE21
GhCLE21.1
GrCLE21
GhCLE22.1
GrCLE22
GaCLE22
GhCLE22.2
GhCLE20.2
GaCLE20
GhCLE20.1
GrCLE20
GrCLE48
GaCLE48

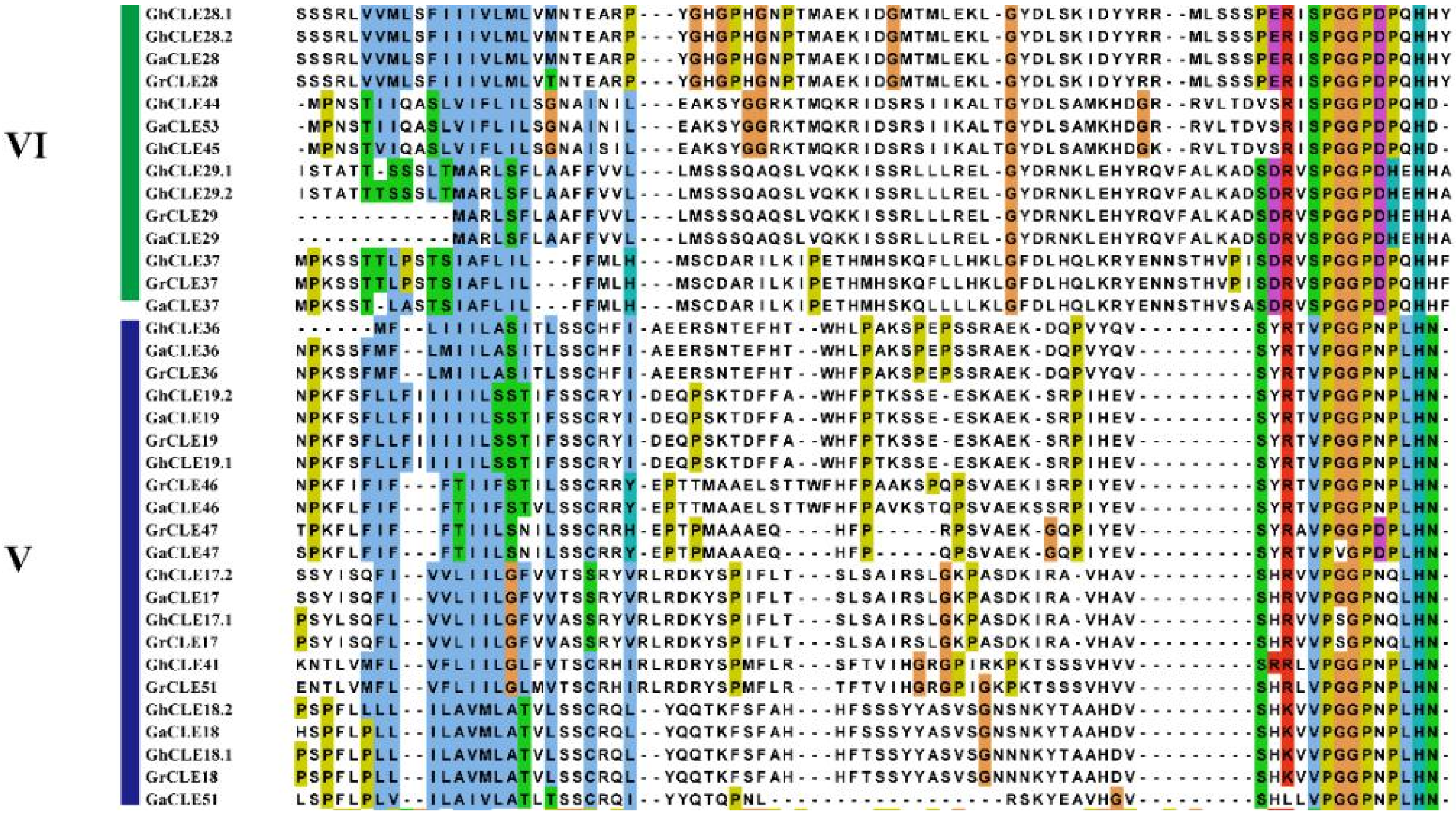
VI
V
GhCLE28.1
GhCLE28.2
GaCLE28
GrCLE28
GhCLE44
GaCLE53
GhCLE45
GhCLE29.1
GhCLE29.2
GrCLE29
GaCLE29
GhCLE37
GrCLE37
GaCLE37
GhCLE36
GaCLE36
GrCLE36
GhCLE19.2
GaCLE19
GrCLE19
GhCLE19.1
GrCLE46
GaCLE46
GrCLE47
GaCLE47
GhCLE17.2
GaCLE17
GhCLE17.1
GrCLE17
GhCLE41
GrCLE51
GhCLE18.2
GaCLE18
GhCLE18.1
GrCLE18
GaCLE51

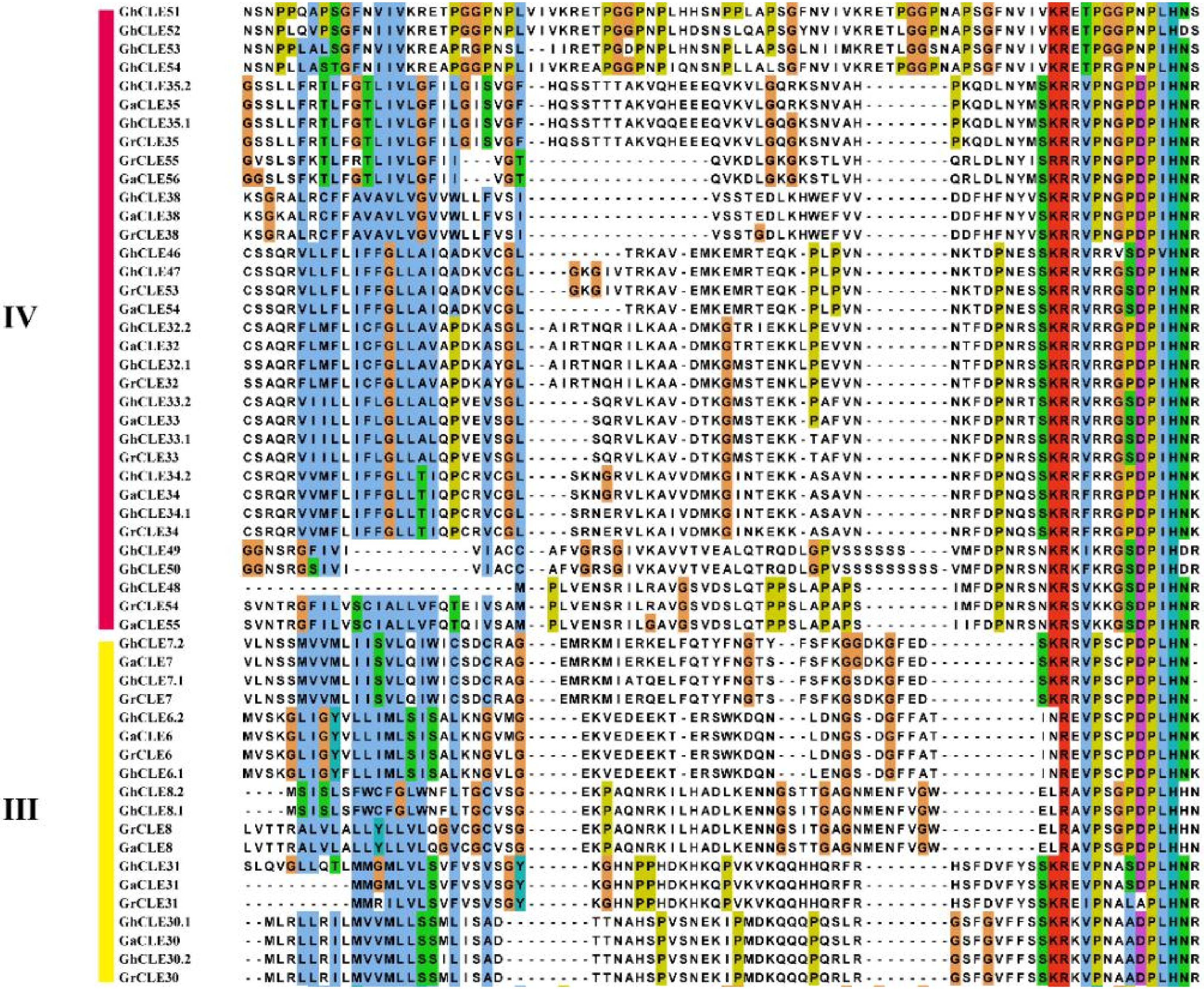
IV
III
GhCLE51
GhCLE52
GhCLE53
GhCLE54
GhCLE35.2
GaCLE35
GhCLE35.1
GrCLE35
GrCLE55
GaCLE56
GhCLE38
GaCLE38
GrCLE38
GhCLE46
GhCLE47
GrCLE53
GaCLE54
GhCLE32.2
GaCLE32
GhCLE32.1
GrCLE32
GhCLE33.2
GaCLE33
GhCLE33.1
GrCLE33
GhCLE34.2
GaCLE34
GhCLE34.1
GrCLE34
GhCLE49
GhCLE50
GhCLE48
GrCLE54
GaCLE55
GhCLE7.2
GaCLE7
GhCLE7.1
GrCLE7
GhCLE6.2
GaCLE6
GrCLE6
GhCLE6.1
GhCLE8.2
GhCLE8.1
GrCLE8
GaCLE8
GhCLE31
GaCLE31
GrCLE31
GhCLE30.1
GaCLE30
GhCLE30.2
GrCLE30

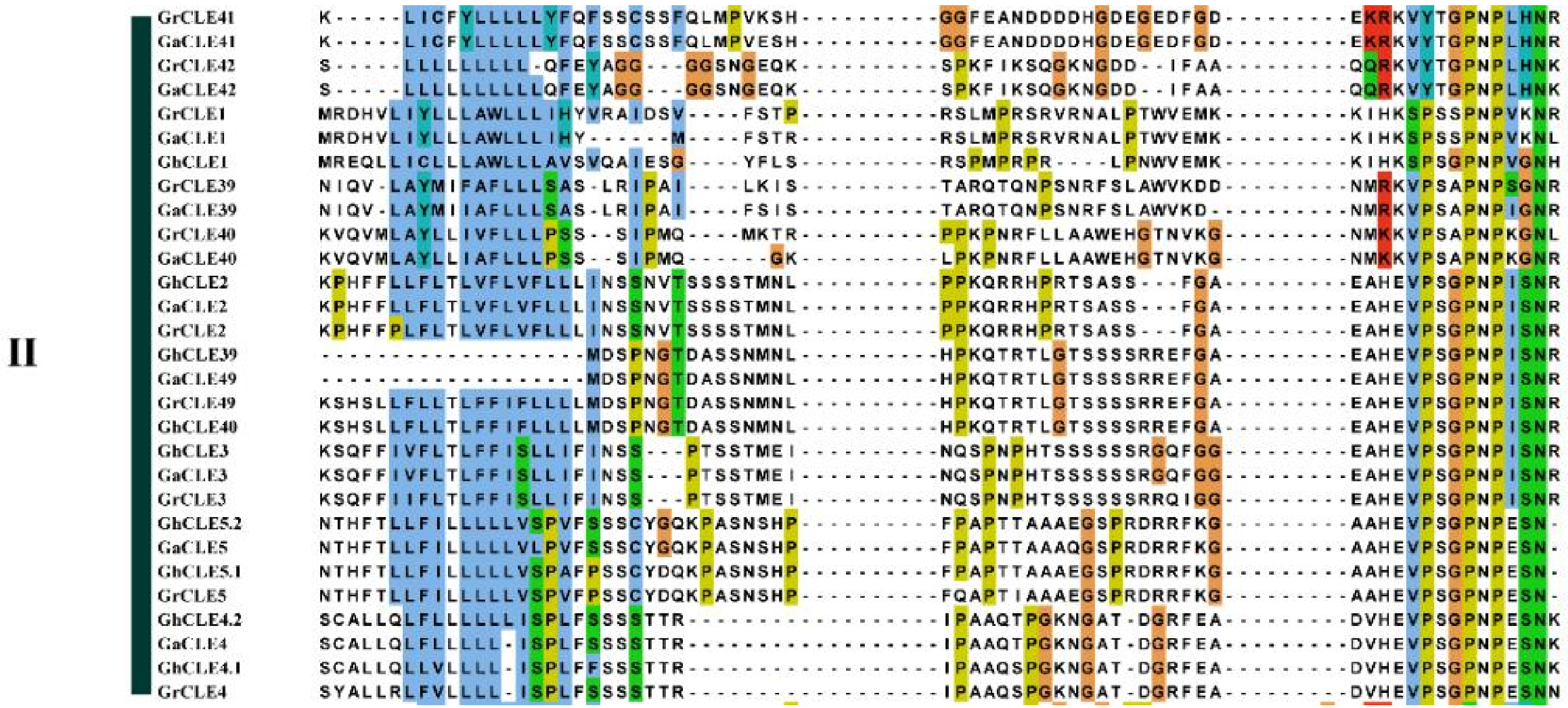
II
GrCLE41
GaCLE41
GrCLE42
GaCLE42
GrCLE1
GaCLE1
GhCLE1
GrCLE39
GaCLE39
GrCLE40
GaCLE40
GhCLE2
GaCLE2
GrCLE2
GhCLE39
GaCLE49
GrCLE49
GhCLE40
GhCLE3
GaCLE3
GrCLE3
GhCLE5.2
GaCLE5
GhCLE5.1
GrCLE5
GhCLE4.2
GaCLE4
GhCLE4.1
GrCLE4

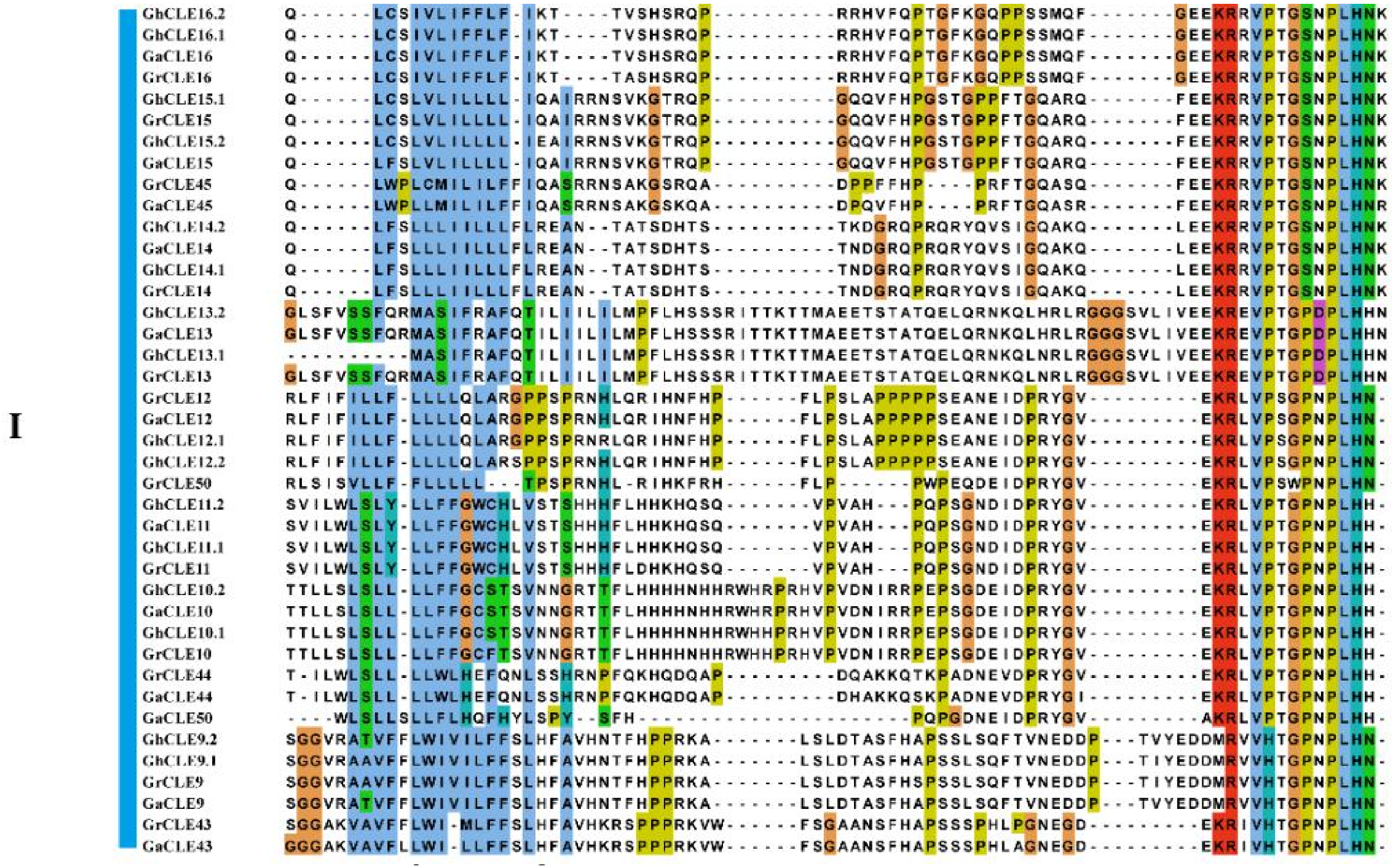


**Supplementary Figure 3**. Multiple sequence alignment of *G.hirsutum*, *G.arboreum*, and *G.raimondii* CLE pre-propeptides was performed using the ClustalX program. The CLE domain contains 14 amino acids, and the leucine-rich region in the signal peptide region is the most conserved in the whole pre-propeptide family.

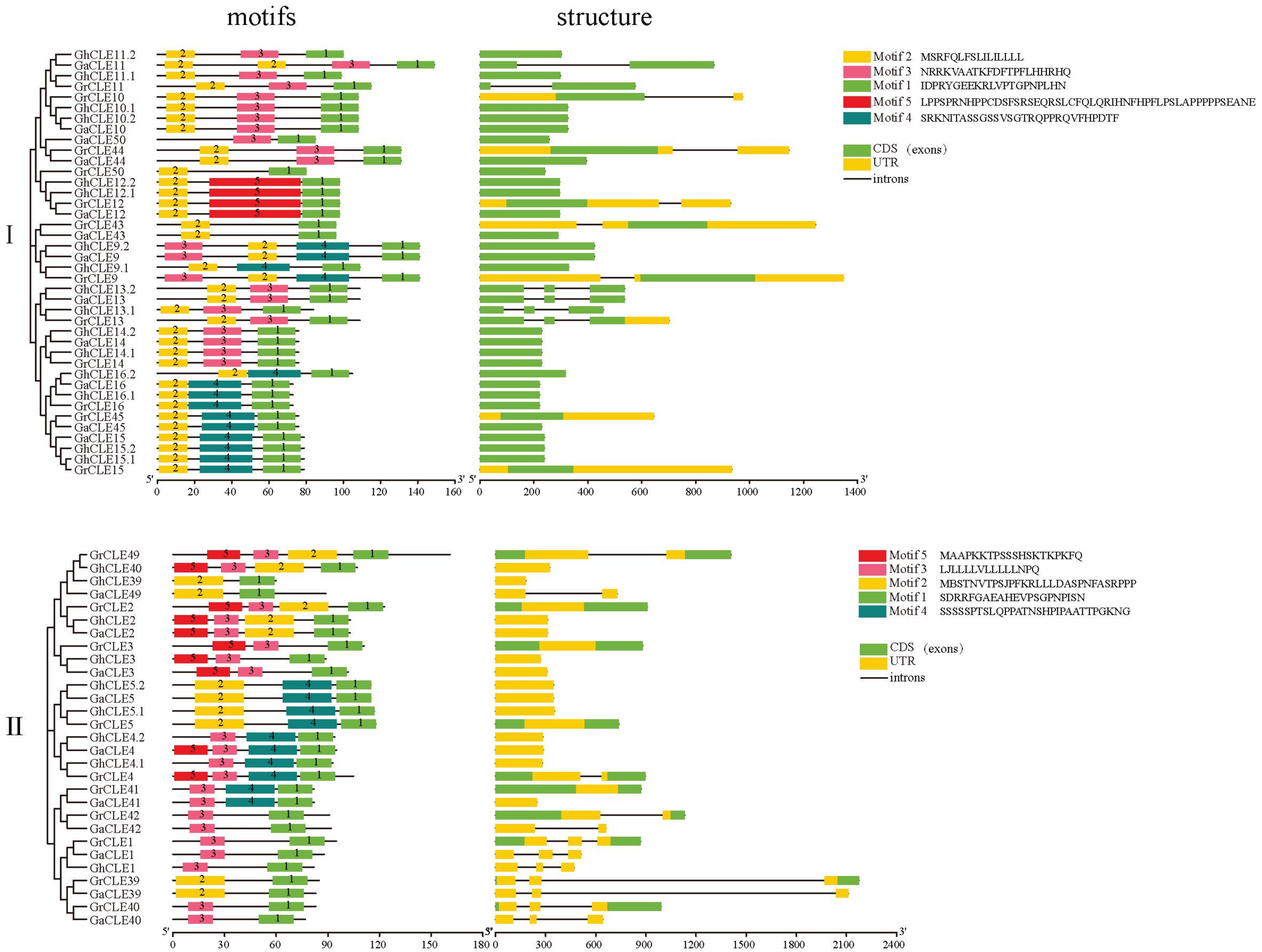
motifs
structure
I
GhCLE11.2
GaCLE11
GhCLE11.1
GrCLE11
GrCLE10
GhCLE10.1
GhCLE10.2
GaCLE10
GaCLE50
GrCLE44
GaCLE44
GrCLE50
GhCLE12.2
GhCLE12.1
GrCLE12
GaCLE12
GrCLE43
GaCLE43
GhCLE9.2
GaCLE9
GhCLE9.1
GrCLE9
GhCLE13.2
GaCLE13
GhCLE13.1
GrCLE13
GhCLE14.2
GaCLE14
GhCLE14.1
GrCLE14
GhCLE16.2
GaCLE16
GhCLE16.1
GrCLE16
GrCLE45
GaCLE45
GaCLE15
GhCLE15.2
GhCLE15.1
GrCLE15
Motif 2 MSRFQLFSLILILLLL
Motif 3 NRRKVAATKFDFTPFLHHRHQ
Motif 1 IDPRYGEEKRLVPTGPNPLHN
Motif 5 LPPSPRNHPPCDSFSRSEQRSLCFQLQRIHNFHPFLPSLAPPPPPSEANE
Motif 4 SRKNITASSGSSVSGTRQPPRQVFHPDTF
CDS (exons)
UTR
introns
5' 0 20 40 60 80 100 120 140 160 3'
5' 0 200 400 600 800 1000 1200 1400 3'
II
GrCLE49
GhCLE40
GhCLE39
GaCLE49
GrCLE2
GhCLE2
GaCLE2
GrCLE3
GhCLE3
GaCLE3
GhCLE5.2
GaCLE5
GhCLE5.1
GrCLE5
GhCLE4.2
GaCLE4
GhCLE4.1
GrCLE4
GrCLE41
GaCLE41
GrCLE42
GaCLE42
GrCLE1
GaCLE1
GhCLE1
GrCLE39
GaCLE39
GrCLE40
GaCLE40
Motif 5 MAAPKKTPSSSHSKTKPKFQ
Motif 3 LJLLLLVLLLLLNPQ
Motif 2 MBSTNVTPSJPFKRLLLDASPNFASRPPP
Motif 1 SDRRFGAEAHEVPSGPNPISN
Motif 4 SSSSSPTSLQPPATNSHPIPAATTPGKNG
CDS (exons)
UTR
introns
5' 0 30 60 90 120 150 180
5' 0 300 600 900 1200 1500 1800 2100 2400 3'

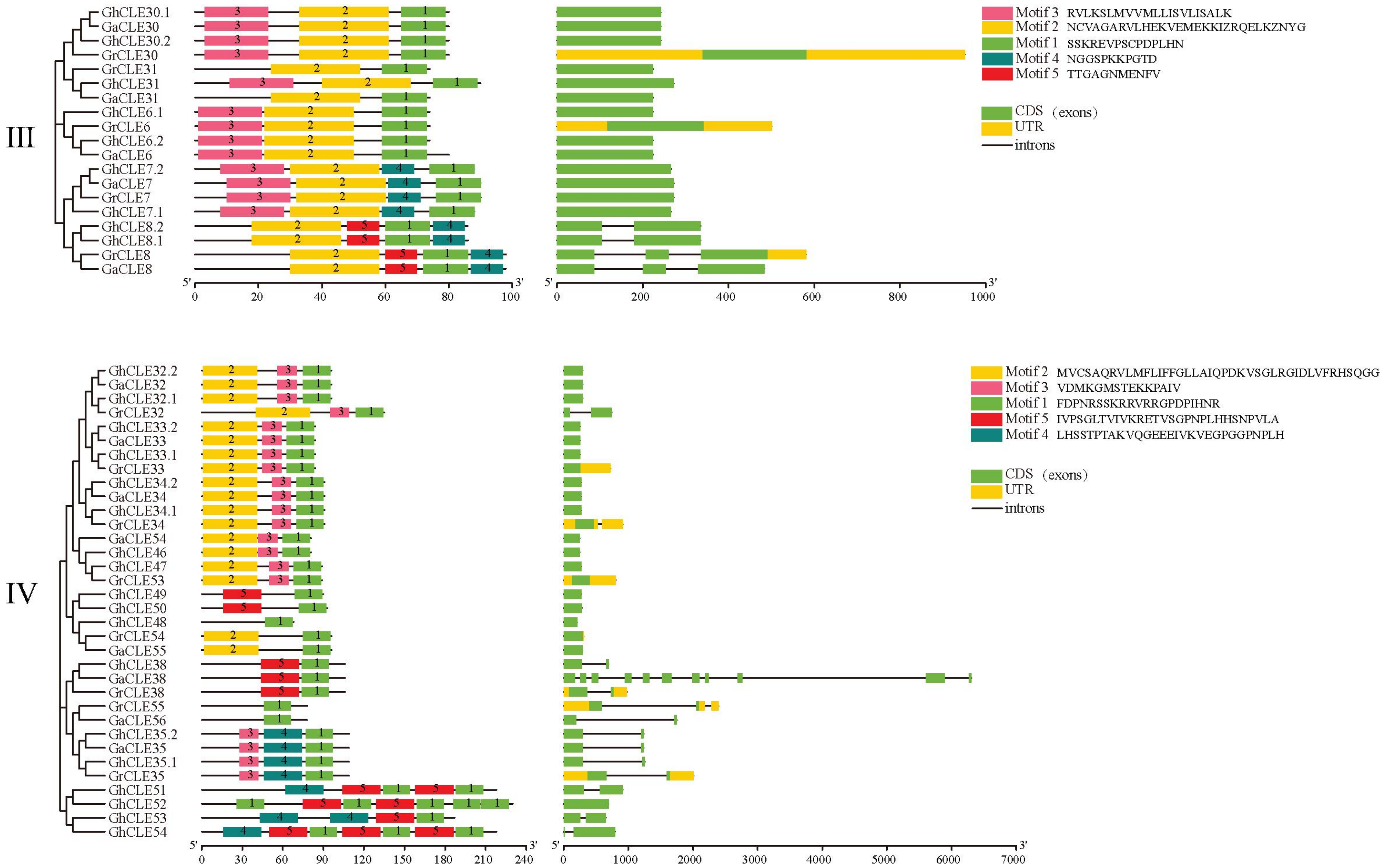
III
GhCLE30.1
GaCLE30
GhCLE30.2
GrCLE30
GrCLE31
GhCLE31
GaCLE31
GhCLE6.1
GrCLE6
GhCLE6.2
GaCLE6
GhCLE7.2
GaCLE7
GrCLE7
GhCLE7.1
GhCLE8.2
GhCLE8.1
GrCLE8
GaCLE8
Motif 3 RVLKSLMVVMLLISVLISALK
Motif 2 NCVAGARVLHEKVEMEKKIZRQELKZNYG
Motif 1 SSKREVPSCPDPLHN
Motif 4 NGGSPKKPGTD
Motif 5 TTGAGNMENFV
CDS (exons)
UTR
introns
5' 3'
0 20 40 60 80 100
5' 3'
0 200 400 600 800 1000
IV
GhCLE32.2
GaCLE32
GhCLE32.1
GrCLE32
GhCLE33.2
GaCLE33
GhCLE33.1
GrCLE33
GhCLE34.2
GaCLE34
GhCLE34.1
GrCLE34
GaCLE54
GhCLE46
GhCLE47
GrCLE53
GhCLE49
GhCLE50
GhCLE48
GrCLE54
GaCLE55
GhCLE38
GaCLE38
GrCLE38
GrCLE55
GaCLE56
GhCLE35.2
GaCLE35
GhCLE35.1
GrCLE35
GhCLE51
GhCLE52
GhCLE53
GhCLE54
Motif 2 MVCSAQRVLMFLIFFGLLAIQPDKVSGLRGIDLVFRHSQGG
Motif 3 VDMKGMSTEKKPAIV
Motif 1 FDPNRSSKRRVRRGPDPIHNR
Motif 5 IVPSGLTVIVKRETVSGPNPLHHSNPVLA
Motif 4 LHSSTPTAKVQGEEEIVKVEGPGGPNPLH
CDS (exons)
UTR
introns
5' 3'
0 30 60 90 120 150 180 210 240
5' 3'
0 1000 2000 3000 4000 5000 6000 7000

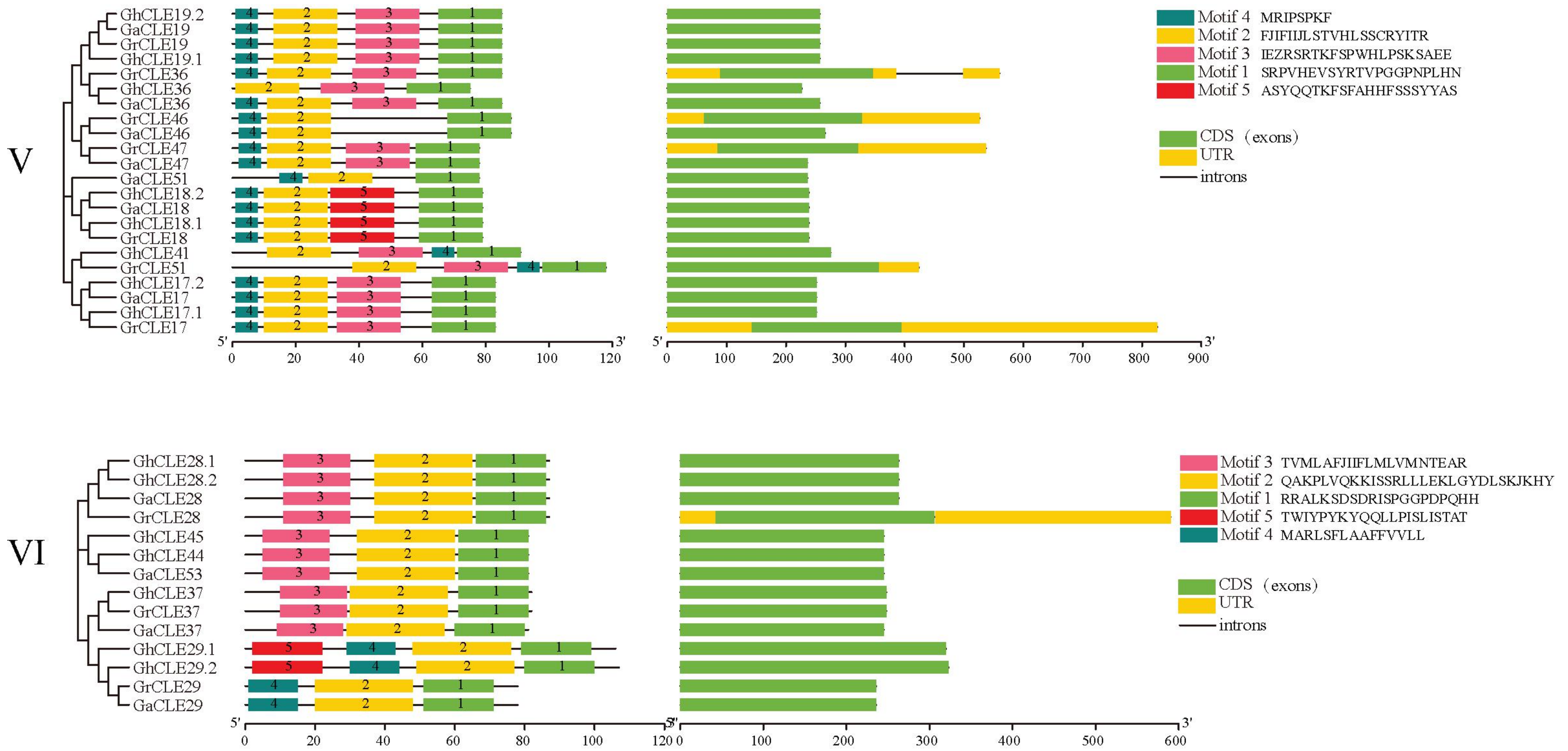

V
GhCLE19.2
GaCLE19
GrCLE19
GhCLE19.1
GrCLE36
GhCLE36
GaCLE36
GrCLE46
GaCLE46
GrCLE47
GaCLE47
GaCLE51
GhCLE18.2
GaCLE18
GhCLE18.1
GrCLE18
GhCLE41
GrCLE51
GhCLE17.2
GaCLE17
GhCLE17.1
GrCLE17
Motif 4 MRIPSPKF
Motif 2 FJIFIIJLSTVHLSSCRYITR
Motif 3 IEZRSRTKFSPWHLPSKSAEE
Motif 1 SRPVHEVSYRTVPGGPNPLHN
Motif 5 ASYQQTKFSFAHHFSSSYYAS
CDS (exons)
UTR
introns
5′ 0 20 40 60 80 100 120 3′
5′ 0 100 200 300 400 500 600 700 800 900 3′
VI
GhCLE28.1
GhCLE28.2
GaCLE28
GrCLE28
GhCLE45
GhCLE44
GaCLE53
GhCLE37
GrCLE37
GaCLE37
GhCLE29.1
GhCLE29.2
GrCLE29
GaCLE29
Motif 3 TVMLAFJIIFLMLVMNTEAR
Motif 2 QAKPLVQKKISSRLLLEKLGYDLSKJKHY
Motif 1 RRALKSDSDRISPGGPDPQHH
Motif 5 TWIYPYKYQQLLPISLISTAT
Motif 4 MARLSFLAAFFVVLL
CDS (exons)
UTR
introns
5′ 0 20 40 60 80 100 120
5′ 0 100 200 300 400 500 600 3′

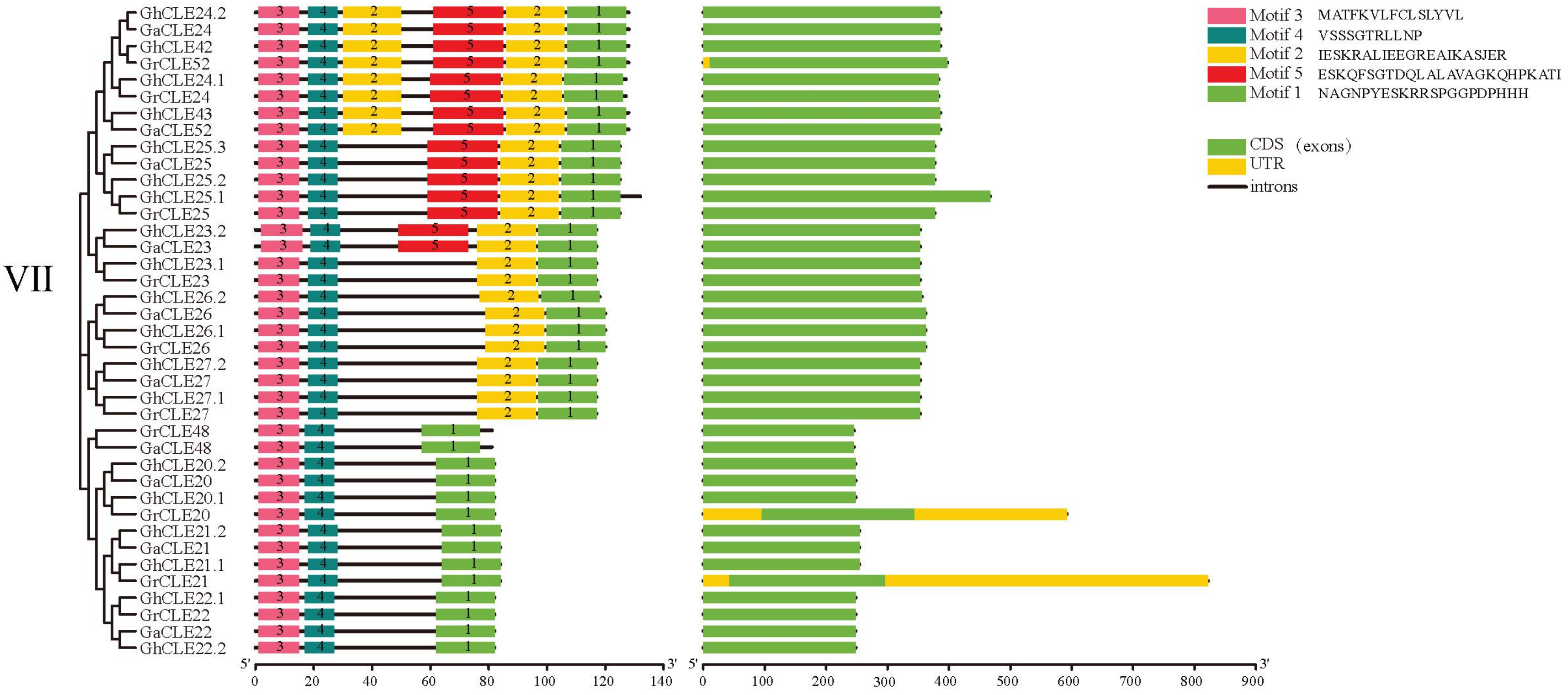


**Supplementary Figure 4.** Gene structure and conserved motifs of seven groups in *G.hirsutum, G.arboreum, and G.raimondii* were illustrated by GSDS 2.0 and MEME. In gene structure diagrams, green boxes standed for exons; black lines for introns; yellow boxes for UTR. Five conserved motifs of each group were identified. Different color boxes with number represented different motifs.
